# Seeing speech: Neural mechanisms of cued speech perception in prelingually deaf and hearing users

**DOI:** 10.1162/IMAG.a.53

**Published:** 2025-06-24

**Authors:** Annahita Sarré, Laurent Cohen

**Affiliations:** Inserm U 1127, CNRS UMR 7225, Sorbonne Universités, Institut du Cerveau, ICM, Paris, France; AP-HP, Hôpital de La Pitié Salpêtrière, Fédération de Neurologie, Paris, France

**Keywords:** deafness, language, functional MRI, cued speech, VWFA, EBA

## Abstract

For many deaf people, lip-reading plays a major role in verbal communication. However, lip movements are by nature ambiguous, so that lip-reading does not allow for a full understanding of speech. The resulting language access difficulties may have serious consequences on language, cognitive and social development. Cued speech (CS) was developed to eliminate this ambiguity by complementing lip-reading with hand gestures, giving access to the entire phonological content of speech through the visual modality alone. Despite its proven efficiency for improving linguistic and communicative abilities, the mechanisms of CS perception remain largely unknown. The goal of the present study is to delineate the brain regions involved in CS perception and identify their role in visual and language-related processes. Three matched groups of participants were scanned during the presentation of videos of silent CS sentences, isolated lip movements, isolated gestures, plus CS sentences with speech sounds, and meaningless CS sentences: Prelingually deaf users of CS, hearing users of CS, and naïve hearing controls. We delineated a number of mostly left-hemisphere brain regions involved in CS perception. We first found that language areas were activated in all groups by both silent CS sentences and isolated lip movements, and by gestures in deaf participants only. Despite overlapping activations when perceiving CS, several findings differentiated experts from novices. The Visual Word Form Area, which supports the interface between vision and language during reading, was activated by isolated gestures in deaf CS users. In contrast, the Bayes factor indicated either weak evidence of no activation or negligible evidence of activation in hearing and control groups. Moreover, the integration of lip movements and gestures took place in a temporal language-related region in deaf users, and in movement-related regions in hearing users, reflecting their different profile of expertise in CS comprehension and production. Finally, we observed a strong involvement of the Dorsal Attentional Network in hearing users of CS, and identified the neural correlates of the variability in individual proficiency. Cued speech constitutes a novel pathway for accessing core language processes, halfway between speech perception and reading. The current study provides a delineation of the common and specific brain structures supporting those different modalities of language input, paving the way for further research.

## 1 Introduction

Writing was invented to allow fleeting utterances the potential of enduring over time. In non-logographic writing systems, this is achieved though the visual coding of speech sounds, with some variation in the sound units being written down, which may be phonemes, syllables, etc. Making sounds visible was also prompted by the need to communicate efficiently with deaf people. Unable to perceive speech through the auditory modality, this population heavily relies on lip-reading when encountering spoken language (Desai et al., 2008;Schorr et al., 2005). However, the phonological information carried by the configuration of the mouth is intrinsically ambiguous, such that the words “bark,” “mark,” and “park” cannot be distinguished on a visual basis. When no effective strategy is implemented to compensate for this limitation and permit good deciphering of phonological contrasts, the resulting language access difficulties may have serious consequences on language, cognitive, and social development (Friedmann & Rusou, 2015;Werker & Hensch, 2015).

In this context, cued speech (CS) was designed by Dr. R. Orin Cornett (Cornett, 1967), with the initial purpose of helping prelingually deaf children improve their reading capacities. Cued speech relies on a set of hand gestures that are designed to counteract the ambiguity of lip-reading, thus giving access to the entire phonological content of speech. As alphabetic scripts are based on a transcription of speech sounds, cued speech improves general linguistic skills of deaf users, notably in reading (Gardiner-Walsh et al., 2020;Trezek, 2017).

**Fig. 1.**
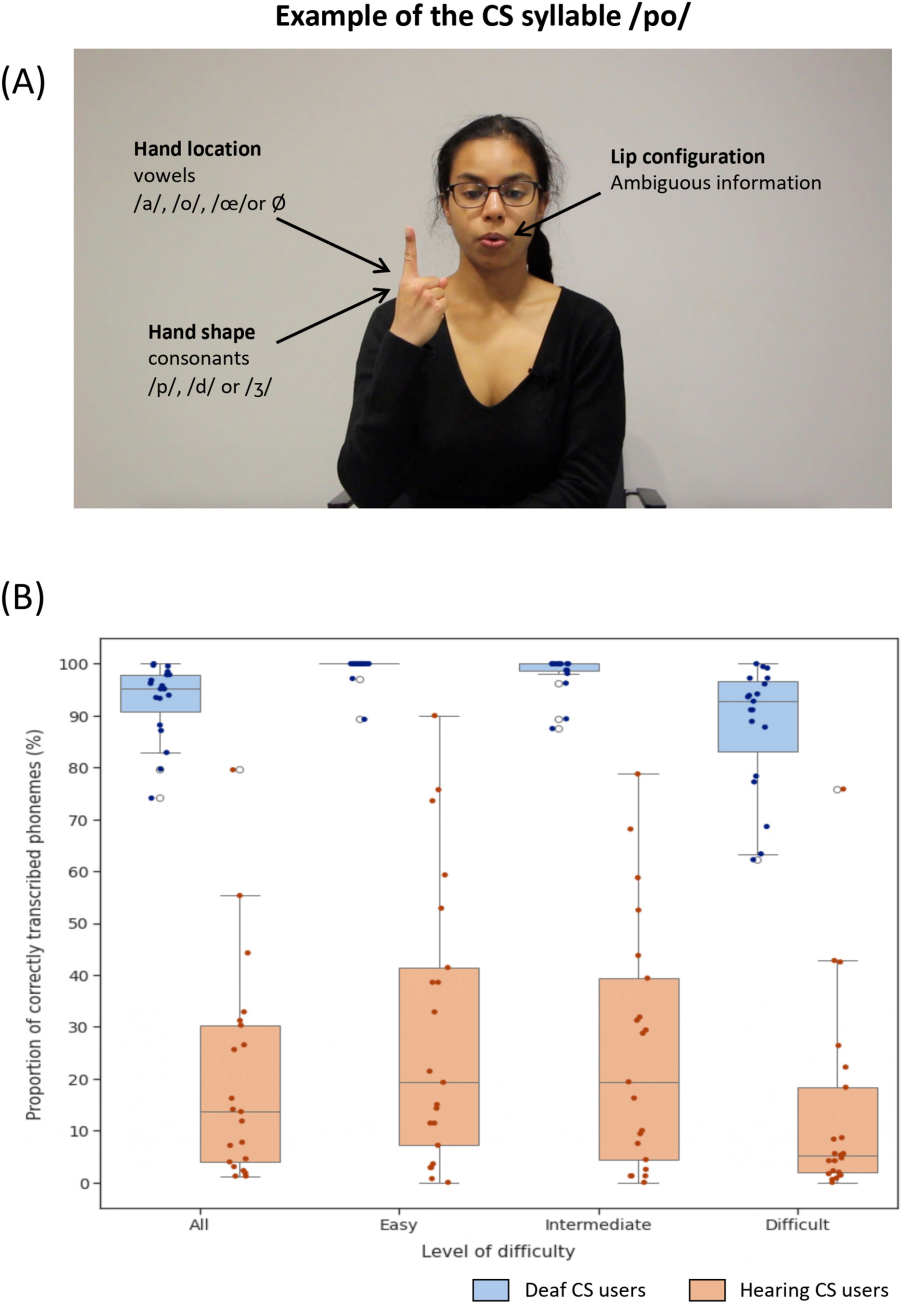
(A) Example syllable from the French cued speech system, extracted from the experimental material. CS systems are designed so that syllables sharing the same lip movement are complemented with different hand gestures, allowing for syllabic identification through the visual modality alone. Hand position specifies vowels, and hand shape specifies consonants; (B) CS comprehension accuracy, as indexed by the percentage of correctly transcribed phonemes, in deaf and hearing CS users, for all sentences and for each level of difficulty. Hearing CS users performed less well, and with larger individual variability, than deaf participants.

In cued speech, words are first decomposed into consonant–vowel (CV) syllables (e.g. “pari” → /pa-ʁi/), sometimes requiring adjustments (e.g. “drakkar” → /d-ʁa-ka-ʁ/ instead of /dʁa-kaʁ/). The identity of each syllable is then conveyed through three cues: the lip movements, the position of the hand, and the shape of the hand (Fig. 1A). The hand assumes one among eight possible shapes (e.g. index extended and other fingers folded), each shape representing approximately three consonant phonemes (e.g. /p/, /d/ and /ʒ/). In parallel, the hand is placed in one among five possible positions relative to the face (e.g. next to the chin), each position representing approximately three vowel phonemes (e.g. /a/, /o/ and /œ/). The system is designed such that two syllables sharing the same lip movements will be supplemented by two different hand gestures, allowing an easy differentiation. Each unique combination of the three cues corresponds to a specific syllable, allowing cued speech to visually represent the complete phonemic content of spoken language.

The original American CS system was adapted to over 65 languages and dialects (International Academy Supporting Adaptations of Cued Speech (AISAC), 2020), and notably in French where the system is referred to as “Langue française Parlée Complétée” (LfPC). CS is typically used in complement with cochlear implants or, less often, hearing aids.

Naturally CS is not the only way for deaf people to acquire language. The dominant alternative is the use of sign languages, which are fully fledged visual languages distinct from spoken languages and permitting a full linguistic development. When communicating through a given sign language, an ancillary fingerspelling system visually similar to CS can typically be used to convey manually the spelling of words from a spoken language, for instance to communicate proper nouns (Fenlon et al., 2017). Importantly, the use of CS and of a sign language are not incompatible. In fact, studies and guidelines suggest that a bilingual education combining a spoken language supplemented by a CS system and a sign language is beneficial for children (Alegria et al., 1999;Colin et al., 2021).

While sign languages have been the object of a vast number of cognitive neuroscience studies (MacSweeney et al., 2008;Trettenbrein et al., 2021), CS systems have received limited attention from this field. To our knowledge, only two studies have been devoted to the brain mechanisms of CS perception, using MRI (Aparicio et al., 2017) and EEG (Caron et al., 2023).Aparicio et al. (2017)presented single words to deaf users of CS in their full CS form, with hand gestures only, and with lip movements only. The same words were presented to naïve hearing controls in audiovisual form, only auditorily, and with lip movements only. They observed identical activation of core language areas in both groups, and proposed that the integration of CS gestures and lip-reading cues takes place in the left occipitotemporal junction, particularly in area MT/V5. They also suggested that manual cues may play a larger role than lip-reading in CS perception, in line with indirect behavioral evidence (Attina et al., 2004,^2005^;Bayard et al., 2014).

Going beyond those early results, the goal of the present study is to answer fundamental questions on CS perception, using fMRI in three matched groups of participants: prelingually deaf users of cued speech, hearing users of cued speech, and naïve hearing controls. We will explore the following issues and assess the associated predictions.

First, what are the sectors of the visual cortex which are processing hand configurations and lip-reading, and do activation patterns differ between groups, reflecting expertise in CS perception? Concerning hand gestures, the key candidates are the visual word form area (VWFA) and the lateral occipitotemporal cortex (LOTC), two specialized high-level visual areas. Following reading acquisition, the VWFA specializes for the recognition of written letters and words, as shown by a host of neuropsychological and imaging studies (Dehaene et al., 2015). As for the LOTC, it is essential for recognizing all sorts of hand gestures including object manipulation or social communication (Bracci et al., 2018;Wurm & Caramazza, 2019). According to “top-down” theories, the specialization of the VWFA results from its predisposition to interface visual shape analysis with language areas, in which case it should intervene not only in reading but also in CS perception (Price & Devlin, 2011). Conversely, if specialization in the occipitotemporal cortex is mainly driven “bottom-up” by the visual features of stimuli, then the recognition of hand gestures in the context of CS should rely on the LOTC. The predictions are quite different regarding lip-reading. The same mouth configurations are used to convey similar phonological information in both visual and cued speech. Therefore, we predict that the same areas should be involved in lip-reading during CS perception in deaf participants than during visual speech perception in the general population.

Second, how does CS input drive language areas, as compared with the usual visual speech? As CS is only an alternative entry code to the same common language, we predict that the activation of the core, modality independent, language areas (Fedorenko et al., 2024) should be the same in deaf CS users perceiving CS sentences, and in hearing participants perceiving speech. This situation would be similar to the activation of language areas by written and by spoken input (Rueckl et al., 2015). However, the two components of CS, when used in isolation, generate distinct predictions. On the one hand, leaving aside possible differences in expertise, pure lip-reading in the absence of gestures and sound should activate language areas similarly in all groups, as this cue is commonly used for comprehension by both deaf and hearing individuals. On the other hand, perceiving only the gestural component of CS, which is meaningless for naïve participants, may possibly activate language areas exclusively in CS users, again with possible modulations by expertise.

Third, language comprehension often relies on the integration of information conveyed through different acoustic dimensions, like in the integration of speech prosody with syntax (Degano et al., 2024), or through different modalities, like in audiovisual speech perception (Hickok et al., 2018;Ross et al., 2022). Similarly in CS, visual cues from hand position, hand shape, and mouth configuration must be combined to uniquely identify the target syllable. We will try to adjudicate between two options. The convergence may potentially take place either in visual cortices, where each syllable would be represented as a complex gestural combination, or more downstream in language areas, where the different cues would converge to select the appropriate phonological syllable. Both types of convergence of codes have been shown to occur in the field of reading. In alphabetic scripts, upper-case and lower-case letters converge in the occipitotemporal visual cortex (Dehaene et al., 2001), while in Japanese the logographic kanji and the syllabic kana scripts converge in left temporal language areas (Nakamura et al., 2005).

Fourth, we will address the potentially complex case of hearing CS users. These participants typically master CS production better than perception, as they use CS to address deaf relatives or people whom they assist, but are rarely addressed to in silent CS. They also generally learn CS as adults. We will, therefore, try to clarify where this group stands with respect to the other two, and whether hearing CS users show important individual variability.

To address these questions, we scanned deaf CS users, hearing CS users, and hearing CS naïve controls during an fMRI experiment where we presented videos of different types of sentences in full or degraded French CS. We also conducted a localizer experiment during which we presented pictures of faces, bodies, written words, tools, and houses in order both to understand the contribution of category-specific visual regions to CS perception, and to identify potential modification of the functional layout of ventral occipitotemporal cortex (VOTC) in CS users. MRI acquisition was preceded by a questionnaire on deafness and language history, and by a pretest evaluating the mastery of CS of deaf and hearing CS users.

## 2 Methods

### 2.1 Participants

We recruited 60 healthy volunteers: 19 prelingually and severely/profoundly deaf CS users (hereafter referred to as the “deaf” group), 21 hearing CS users (“hearing” group), and 20 hearing CS naïve controls (“control” group) with no knowledge of CS. Participants were 18–65 years old French native speakers. All participants were right handers according to the Edinburgh inventory (Oldfield, 1971) except for two left-handed deaf participants and one ambidexter hearing user. They had no history of neurological or psychiatric disorders, and all had normal or corrected to normal vision. They did not present contra-indications to MRI, which constituted a particular challenge as deaf CS users typically carry an MRI-incompatible cochlear implant. Only participants with no or removable hearing aids were recruited. For the two groups of CS users, CS comprehension and production were both assessed before the MRI session. For the reason discussed above, deaf participants were required to have a good level in CS comprehension, and hearing participants to master CS production. Recruitment of CS users was done through social networks and mailing lists of CS associations.

Before the MRI session, the participants were asked to fill an initial questionnaire at home containing general demographic questions for all three groups of participants. Deaf CS users were asked additional questions focusing on the etiology and severity of deafness, history of language acquisition, hearing aids, and on the daily use of CS. Hearing CS users answered questions on the learning and use of CS in daily life.

Deaf participants were severely (n = 2), profoundly (n = 16), or totally (n = 1) deaf. All were pre-linguistically deaf, including 16 out of 19 congenital deafness. All reported standard ages of reading acquisition (5.15 ± 1.01 years old), with average to good current reading capacities. Hearing users and controls had normal hearing, except for one hearing CS user who had moderate deafness. The three groups were matched in age (deaf: 35.26 ± 8.12 yo; hearing: 35.52 ± 11.31 yo; controls: 34.35 ± 10.72 yo) and education level (university degree: deaf: n = 17; hearing: n = 19; controls: n = 18). The gender ratio was similar among the three groups (deaf: 12 F; hearing: 16 F; controls: 13 F; χ²(1) = 0.937, p > 0.05). CS users differed in their age of CS learning (deaf: 2.84 ± 1.71 yo; hearing: 24.95 ± 10.49 yo). All but two users practiced CS at least once a month, with more hearing participants practicing daily or several times a week (deaf: n = 11; hearing: n = 16). The two remaining participants were proficient deaf early CS users and scored high on the pretest. Moreover, 15 deaf and 13 hearing users declared having knowledge on French Sign Language, with a later age of learning than for CS (deaf: 15.67 ± 9.24 yo; hearing: 24.61 ± 10.98 yo) and varying self-declared levels in comprehension and production but a rather frequent use by deaf participants (12 using French Sign Language at least once a month).

All information was provided in written form identically to the three groups of participants. The research was approved by the institutional review board “Comité de Protection des Personnes” Est-III (N° CPP 20.11.05). All participants provided informed written consent in accordance with the Declaration of Helsinki.

### 2.2 Behavioral assessment

The basic principles of CS were explained to controls, and we familiarized them with CS by showing them a series of CS syllables while asking them to detect repetitions. The proficiency of CS users in CS comprehension and production was assessed before the MRI session.

Comprehension was tested through a short dictation test, where the participants had to write down 12 CS sentences presented as silent videos. Each sentence was presented twice, and participants had to respond after each viewing. Responses were assessed by computing for each sentence the percentage of correctly transcribed phonemes. Spelling mistakes were disregarded as long as they transcribed the correct phoneme. Results were then averaged across sentences. CS production was tested by asking participants to transpose 12 written sentences into CS. Sufficient accuracy and fluency of responses were checked by the experimenter.

For both tests, sentences were distributed into three levels of difficulty. “Easy” sentences were short, used the present tense, included only frequent and semantically predictable words, and CV syllables. “Intermediate” sentences were short, used various tenses, and included frequent but less predictable words and one complex syllabic pattern (V-CCV, V-CVC, or VC-CV) (Alegria et al., 1999). “Difficult” sentences were longer, used various tenses, included less frequent and less predictable words, and one complex syllabic pattern.

### 2.3 Brain activation during cued speech perception

The experiment included five conditions: (1) sentences in full CS (with sound), (2) sentences in full CS (silent), (3) sentences in lip-reading only (silent), (4) sentences with only the gestural part of CS (silent), (5) meaningless pseudo-sentences in full CS (silent), as well as baseline periods with a fixation cross which was absent during the presentation of stimuli.

For each condition, we created 16 different stimuli. Each of these sentence or pseudo-sentence included 19 CS gestures, and lasted about 5 s. All the videos showed the upper body and face of the coder (Fig. 1A) on a 24° x 13.5° screen, plus the hands in conditions involving gestures. Pseudo-sentences were derived from a subset of the real sentences from conditions 1–4, in which open-class words were modified into pseudo-words.

Two stimuli from the same condition were always presented consecutively. Each of such pair started with a 0.2 s image of the background without the coder, followed by a 0.2 s smooth transition, then the two stimuli separated by a 0.2 s transition. The pair ended with a 0.2 s transition and 0.2 s of the background image, for a total duration of 13 s per pair. Each stimulus was used once, resulting in eight pairs per condition.

Moreover, 8 baseline periods of 13 s each were displayed. They consisted of a black fixation cross at the location of the coder’s chin, on an image of the background.

The 40 pairs (8 for each of the 5 conditions) and 8 baseline periods were combined in 2 pseudo-random orders, such that no condition could be presented more than twice in a row, and that 2 baseline periods never occurred consecutively. Half the subjects were randomly presented with each order. The experiment had a total duration of about 11 min, and began and ended with additional baseline periods.

Material for the pretest and the experiment were recorded at the Paris Brain Institute with a professional CS coder and edited using iMovie. Stimuli presentation was done using Psychtoolbox Version 3 (Brainard, 1997) in MATLAB R2019b.

Participants were asked to pay attention to the stimuli and to understand them as much as they could. After the scanning session, the experimenter asked participant to indicate on a 1–5 scale their degree of focus during the experiment. The three groups did not differ (F(2,57) = 2.12, p = 0.13).

### 2.4 Functional localizer: brain activation during visual objects perception

In order to functionally localize regions of interest, static images were presented to participants, distributed among 5 visual object categories, each represented by 20 pictures: faces, bodies, French words, houses, and tools (for a full description of stimuli seeZhan et al., 2023).

Further methods and results specific to the functional localizer are included in theSupplementary Materials, along with a dedicated discussion.

### 2.5 Image acquisition and preprocessing

MRI data were acquired on a Siemens 3T Prisma system at the CENIR imaging center (Paris Brain Institute), using 20 (main experiment) and 64 (functional localizer) channels head coils. To record fMRI data, we used the multi-echo multi-band approach to have high SNR and coverage of the areas sensitive to signal dropout, particularly the lower part of the temporal lobes, while keeping good spatiotemporal resolution. Sequence parameters were TR/TEs/FA = 1660 ms/14.2 ms, 35.39 ms, 56.58ms/74°, isotropic voxel size of 2.5 mm, 60 slices, acceleration factors were multi-band=3, and iPat(GRAPPA)=2. In most participants, pulse oximeter and respiration belt signals were recorded and used for denoising of the data. The anatomical image was a 3DT1 with 1 mm isotropic voxels, using an MPRAGE sequence.

The anatomical image was segmented and normalized to the MNI space using CAT12 (Gaser, 2020). Minimal preprocessing was then conducted with AFNI library (Cox, 1996;Cox & Hyde, 1997) using afni_proc.py wrapper to perform temporal despiking (despike), slice timing correction (tshift), and movement correction (volreg). The volume registration was computed on the first (shortest) echo, and applied to all echoes, where the target for the registration was the MIN_OUTLIER volume, corresponding to the volume with minimal movement. The three echoes were optimally combined with TEDANA library (The Tedana Community et al., 2021). First, a T2* map was computed using all echoes, then the echoes were combined with a weighted sum where the weights were a combination of TE and T2*. All subsequent steps used SPM12. Optimally combined volumes were coregistered to the anatomical scan, and normalized to MNI space using the deformation field computed for the anatomical scan. Finally, data were smoothed using a Gaussian kernel of 4 mm FWHM.

A set of noise regressors were derived from the preprocessing using TAPAS/PhysIO (Kasper et al., 2017), derived from cardiac and respiratory recordings (RETROICOR), cardiac recordings (HRV), respiratory recordings (RVT), white-matter and CSF time series, and PCA to reduce their dimensionality (Noise ROI), realignment parameters, their derivatives, and squared parameters and derivatives, and stick regressors to scrub data from volumes with FD > 0.5 mm.

### 2.6 Statistical analyses

For single-subject analyses, General Linear Models (GLMs) were created and estimated for each experiment and participant using SPM12, with a regressor for each experimental condition, plus regressors for targets and motor responses for the functional localizer, as well as movement and physiological regressors. For group-level analyses, individual contrast images were entered in t-test models for the different comparisons of interest. Unless stated otherwise, the statistical threshold was set to p < 0.001 voxel-wise, and q < 0.05 cluster-wise FDR corrected for multiple comparisons across the whole brain. Effect sizes reported in the table were computed using the Measures of Effect Size (MES) toolbox (Gerchen et al., 2021;Hentschke & Stüttgen, 2011) for SPM12. Cerebellar activations are only reported in the tables.

Some analyses of the data of the CS perception experiment were carried out in individual VOTC regions of interest (ROI) defined on the basis of the functional localizer. To define those ROIs, we first identified the peak coordinate of the following group-level contrasts: the conjunctions of activations in all three groups of Faces > Other categories to identify the Fusiform Face Area (FFA), of Bodies > Other categories to identify the Extrastriate Body Area, and of Words > Other categories to identify the VWFA. For each contrast, we selected a peak in each hemisphere, except for the strongly left-lateralized VWFA, for which we selected the symmetric right-hemisphere coordinates. Around each peak, we defined a sphere of 8 mm radius. Finally, within each of those spheres, we identified the individual local peak activation in the corresponding contrast. Individual 4 mm radius spheres centered on those peaks were used as ROIs in which data from the main experiment were sampled. For analysis, we used the one-sample t-test function from the pingouin Python module and interpreted the Bayes factor following theLee and Wagenmakers (2014)guideline.

## 3 Results

### 3.1 Behavioral assessment of cued speech comprehension

The deaf and hearing CS users were presented with silent CS sentences and asked to write them down. Each sentence was presented twice, and two transcriptions were required.

Averaging both answers, deaf CS users responded accurately (in each sentence, 92.91 ± 7.27% of phonemes were correctly transcribed), showing little variation across subjects (Fig. 1B). The performance of hearing CS users was on average much lower (19.72 ± 20.56%; Mann–Whitney U = 398, p < 0.005, rank-biserial measure of effect size r_rb_= 0.99), and more heterogeneous (Levene’s test for variance comparison: F = 7.07, p < 0.05). The superiority of deaf over hearing CS users prevailed separately for the three levels of sentence difficulty (easy: U = 398, p < 0.005, r_rb_= 0.99; medium: U = 399, p < 0.005, r_rb_= 1.00; hard: U = 396, p < 0.005, r_rb_= 0.98).

Neither group showed a significant difference between the “Easy” and the “Intermediate” conditions (p > 0.05), while comparing “Intermediate” and “Difficult” conditions showed a significant effect in the deaf (U = 314.5, p < 0.005, r_rb_= 0.74) but not in the hearing group. Both groups showed a significant difference between the “Easy” and “Difficult” conditions (deaf: U = 335.5, p < 0.005, r_rb_= 0.86; hearing: U = 301.5, p < 0.05, r_rb_= 0.37).

Looking for differences between the first and second responses, we observed that, in the deaf group, the second attempt was significantly better than the first, for all sentences together (U = 90.5, p < 0.01, r_rb_= -0.50), and for hard sentences separately (U = 96, p < 0.05, r_rb_= -0.47). No difference between attempts was found in the hearing group.

In summary, as anticipated in our fourth question, comprehension performance was excellent and homogeneous in deaf participants, and much lower and less homogeneous in hearing users of CS.

### 3.2 Brain activations during cued speech perception

In order to identify regions activated during the perception of CS, we presented silent CS sentences (henceforth called the “Sentences” condition), silent sentences with only lip-reading cues (“Lip-reading” condition), silent sentences with only gestural cues (“Gestures” condition), CS sentences presented with the corresponding speech sound (“Audible sentences” condition), and silent CS sentences made up of meaningless words (“Pseudo-sentences” condition).

#### 3.2.1 Activation by silent sentences in cued speech

**Fig. 2.**
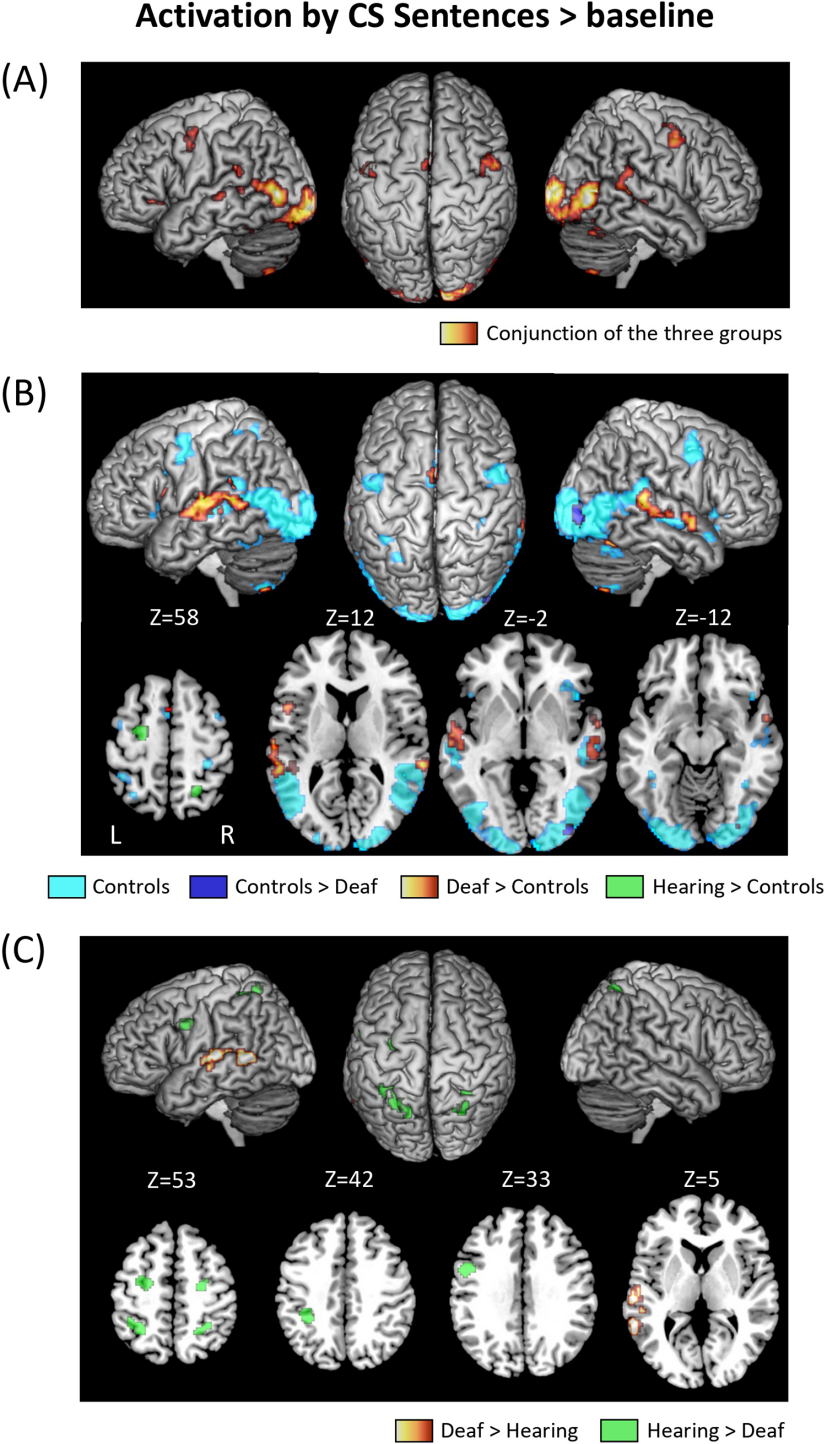
Activation by CS Sentences > baseline. (A) Activations common to the three groups; (B) Activations in Controls, in Controls > Deaf, in Deaf > Controls and in Hearing > Controls; (C) Activations in Deaf > Hearing and Hearing > Deaf.

In order to delineate the overall set of regions activated during CS perception, we first compared Sentences > baseline (Fig. 2;Table 1; see also individual group activations inSupplementary Fig. 1).

**Table 1.** Sentences > baseline.

	Conjunction 3 groups					Deaf > Controls					Deaf > Hearing					Hearing > Deaf					Controls > Deaf					Hearing > Controls				
Region	x	y	z	Z	g	x	y	z	Z	g	x	y	z	Z	g	x	y	z	Z	g	x	y	z	Z	g	x	y	z	Z	g
L Calcarine sulcus	-12	-101	-7	7.66	0.30																									
R Calcarine sulcus	12	-101	3	> 8	0.29																									
L Inferior occipital gyrus	-20	-96	-7	7.23	0.09																									
R Inferior occipital gyrus	22	-96	3	6.99	0.24																40	-86	-4	4.15	1.35					
L LOTC	-48	-71	3	6.83	0.13																									
R LOTC	48	-68	-2	> 8	0.01																									
L Fusiform gyrus	-42	-44	-20	5.78	0.02																									
R Fusiform gyrus	40	-48	-14	4.66	0.24																									
L aSTS/STG	-65	-26	3	3.62	0.90						-60	-11	3	3.39	0.99															
R aSTS/STG	52	-16	-7	3.9	0.33	58	2	-7	4.28	1.48																				
L pSTS/STG	-48	-46	10	5.03	0.26	-58	-41	10	5.09	1.81	-52	-34	8	4.46	1.50															
R pSTS/STG	50	-36	6	5.75	0.72	65	-36	8	4.84	1.72																				
L Middle temporal gyrus											-62	-48	6	4.17	1.39															
L Supramarginal gyrus	-45	-38	26	3.87	0.46																									
L IFG (pars opercularis)						-50	12	13	4.16	1.46																				
L IFG (pars orbitalis)	-38	29	-2	3.53	0.13																									
L Precentral gyrus	-42	-4	50	4	0.17											-50	4	30	4.63	1.56										
R Precentral gyrus	50	2	46	4.27	0.05																									
L/R SMA	-2	6	56	3.72	0.11	-2	4	68	3.76	1.16																				
L Inferior parietal lobule																-35	-38	43	5.48	2.00										
L IPS																-20	-58	58	4.87	1.70										
R IPS																32	-46	48	4.73	1.59						20	-58	63	3.6	1.08
L FEF																-28	-6	56	5.39	2.00						-22	-11	56	4.88	1.56
R FEF																28	-6	46	4.76	0.56										
L Cerebellum	-10	-74	-44	4.05	0.14																									
	-30	-66	-54	3.54	0.15																									
R Cerebellum	30	-64	-57	4.4	0.58	28	-66	-57	4.37	1.41																				
	12	-76	-44	4.21	0.42	35	-61	-24	5.37	1.48																				
	42	-58	-30	4.47	0.34																									
	28	-61	-27	3.86	0.33																									

Hemisphere and anatomical regions, MNI coordinates, Z score, and Hedges’ g effect size of peak activations. Voxel-wise threshold p < 0.001, cluster-wise threshold p < 0.05 FDR-corrected over the whole brain. The contrast Controls > Hearing (masked by Controls) showed no significant activation.

L = left; R = right; a = anterior; p = posterior; LOTC = lateral occipitotemporal cortex; STS = superior temporal sulcus; STG = superior temporal gyrus; IFG = inferior frontal gyrus; SMA = supplementary motor area; IPS = intraparietal sulcus; FEF = frontal eye field.

The conjunction of this contrast across the three groups showed common activation (Fig. 2A) (1) in bilateral visual regions, including the occipital poles, lateral and inferior occipital cortex, and fusiform gyri; (2) in language-related areas, including the left-hemispheric inferior frontal gyrus (IFG) left supramarginal gyrus (SMG), and supplementary motor area (SMA); and the bilateral superior temporal sulcus (STS), posterior superior temporal gyrus (pSTG), precentral gyrus, and cerebellum.

We then compared CS users with controls (Fig. 2B). First, the comparison of Deaf participants > Controls (masked by Deaf > baseline) showed activation in the left IFG, the bilateral STS/STG (with a more extended activation in the left hemisphere), and the SMA. The opposite contrast of Controls > Deaf (masked by Controls > baseline) only showed right inferior occipital activation. Second, the contrast of Hearing > Controls (masked by Hearing > baseline) activated the left frontal eye field (FEF) and the right intraparietal sulcus (IPS). The opposite contrast of Controls > Hearing (masked by Controls > baseline) showed no significant activation.

We then compared the two groups of CS users (Fig. 2C). The contrast of Deaf > Hearing (masked by Deaf > baseline) activated the left STG and middle temporal gyrus (MTG), plus the right pSTS just below the threshold for cluster extent (26 voxels). The opposite comparison of Hearing > Deaf (masked by Hearing > baseline) showed activation in the bilateral FEF and IPS regions already partially present in the Hearing > Controls comparison, in the left precentral gyrus, plus the right inferior occipital cortex at a lower voxel-wise threshold (p < 0.01).

In summary, we found activation in vision- and language-related areas common to all groups, plus three differences among groups. First, relevant to our second question on the core language areas, there was stronger activation in users of CS as compared with controls, in frontal and temporal language areas in the deaf group, and in temporal regions only in the hearing group. Second, relevant to the fourth question on the specificities of hearing CS users, there was stronger activation in a bilateral frontoparietal IPS/FEF network in the hearing than in both the deaf and the control groups. Third, activation was stronger in both the hearing and control groups than in deaf participants in the right inferior occipital cortex, the only difference among groups observed in the visual cortex.

The fact that controls activated language areas, albeit more weakly than deaf participants, may come as a surprise. In the absence of any comprehension of the gestural code by controls, lip-reading appeared as the likely explanation, which we assessed in the following analyses.

#### 3.2.2 Activation by lip-reading and by gestures

**Fig. 3.**
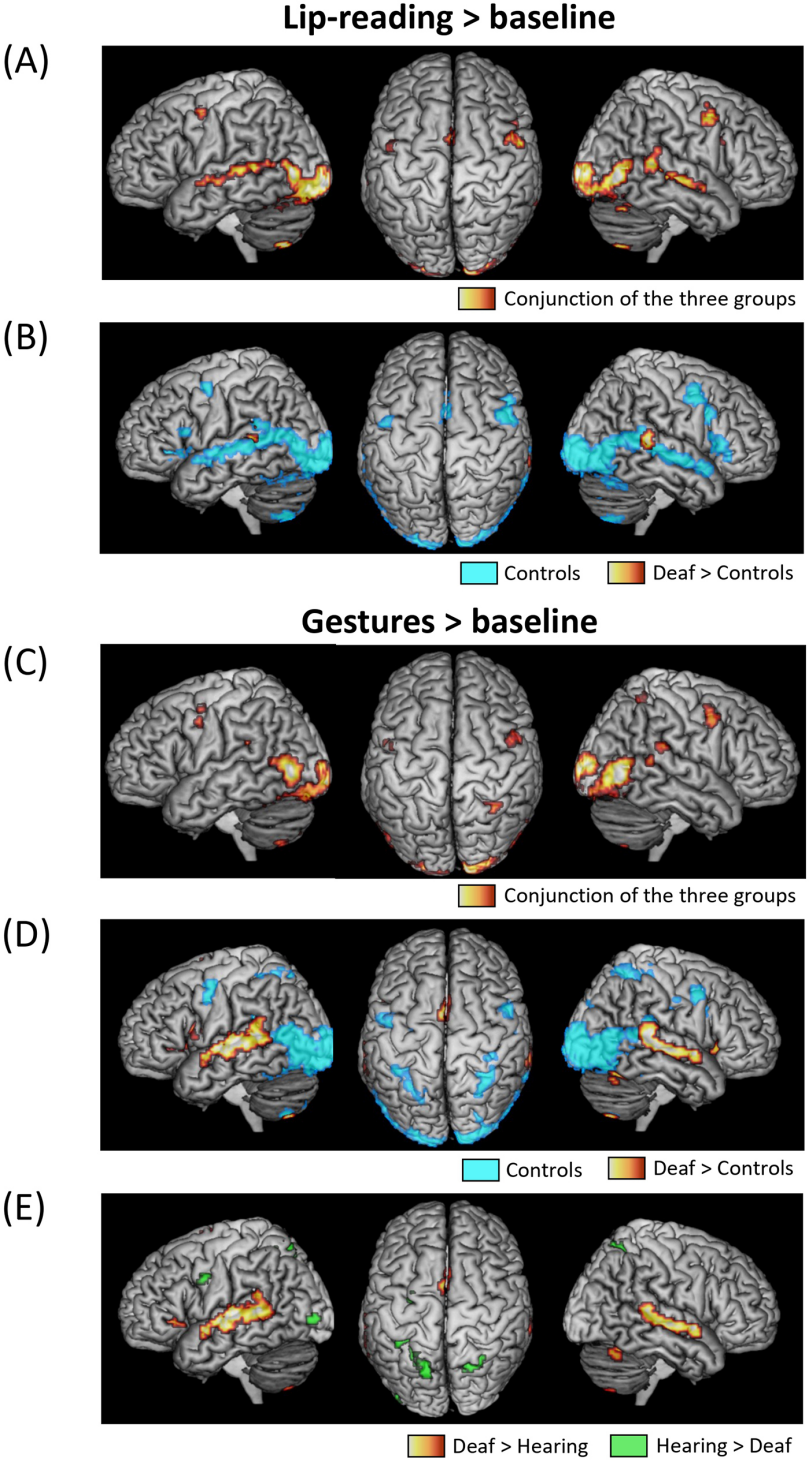
Activation by Lip-reading and Gestures > baseline. (A) Lip-reading > baseline: Activations common to the three groups; (B) lip-reading > baseline: Activations in Controls and in Deaf > Controls; (C) Gestures > baseline: Activations common to the three groups; (D) Gestures > baseline: Activations in Controls and in Deaf > Controls; (E) Gestures > baseline: Activations in Deaf > Hearing and Hearing > Deaf.

We then contrasted conditions with only partial CS information (Lip-reading or Gestures) minus baseline (Fig. 3;Supplementary Figs. 2–3;Supplementary Tables 1–2), in order to determine (i) whether such degraded information was sufficient to activate language areas and the IPS/FEF attention-related network, (ii) whether distinct parts of the visual cortex were preferentially involved in the processing of lip-reading and gestures, and (iii) how groups differed in those respects.

##### 3.2.2.1 Lip-reading

The conjunction of lip-reading > baseline across the three groups activated a large subset of the regions activated by Sentences > baseline: the bilateral occipital poles, inferior occipital cortex, lateral occipital cortex, and fusiform gyrus; plus the bilateral STS/STG, precentral gyrus, and SMA (Fig. 3A). Note that all three groups also showed activation in the left IFG, with only partial overlap, explaining why it did not survive in the conjunction analysis. Pairwise comparisons across groups only showed stronger activation in Deaf > Controls (masked by Deaf > baseline) in the bilateral pSTG/STS (Fig. 3B).

Thus, in agreement with our second prediction, lip-reading activated language areas in all groups including controls, and more so in deaf participants.

##### 3.2.2.2 Gestures

The conjunction of Gestures > baseline in the three groups activated the same occipital and fusiform regions as Lip-reading and Sentences, plus the bilateral posterior tip of the STG as it joins the SMG, the bilateral postcentral and precentral gyri, and the right IPS (Fig. 3C). In deaf participants only, Gestures induced extensive activation of language areas (Supplementary Fig. 3A).

Accordingly, the comparisons of Deaf > Controls (masked by Deaf > baseline) showed activations along the bilateral STG/STS, in the left IFG and in the bilateral insula and SMA (Fig. 3D). The comparison of Deaf > Hearing (masked by Deaf > baseline) showed the same pattern minus the left IFG and the right insula (Fig. 3E).

The comparison of Hearing > Deaf (masked by Hearing > baseline) showed enhanced activations in the same bilateral IPS, left FEF, and precentral regions as observed for Sentences, plus the left inferior and middle occipital cortex (Fig. 3E). Note that controls also activated the IPS/FEF regions, at a level not significantly differing from the deaf group. The other pairwise comparisons between groups showed no differences. Noticeably, Gestures did not activate language areas in hearing more than in controls.

Thus gestures activated language areas only in deaf CS users. Moreover, the IPS/FEF, which were absent from lip-reading activations, were involved in the perception of gestures, more weakly in the controls and deaf groups, and more strongly in hearing users of CS.

##### 3.2.2.3 Comparison between Lip-reading and Gestures

We then contrasted Lip-reading > Gestures (masked by Lip-reading > baseline), and Gestures > Lip-reading (masked by Gestures > baseline) (Supplementary Tables 3–4).

###### 3.2.2.3.1 Visual areas

Regarding our first question, the previous analyses showed that all types of stimuli activated the same sectors of the visual system, with few differences in occipital regions. By comparing Lip-reading and Gestures, we looked for regions more engaged in the processing of one or the other type of cue.

**Fig. 4.**
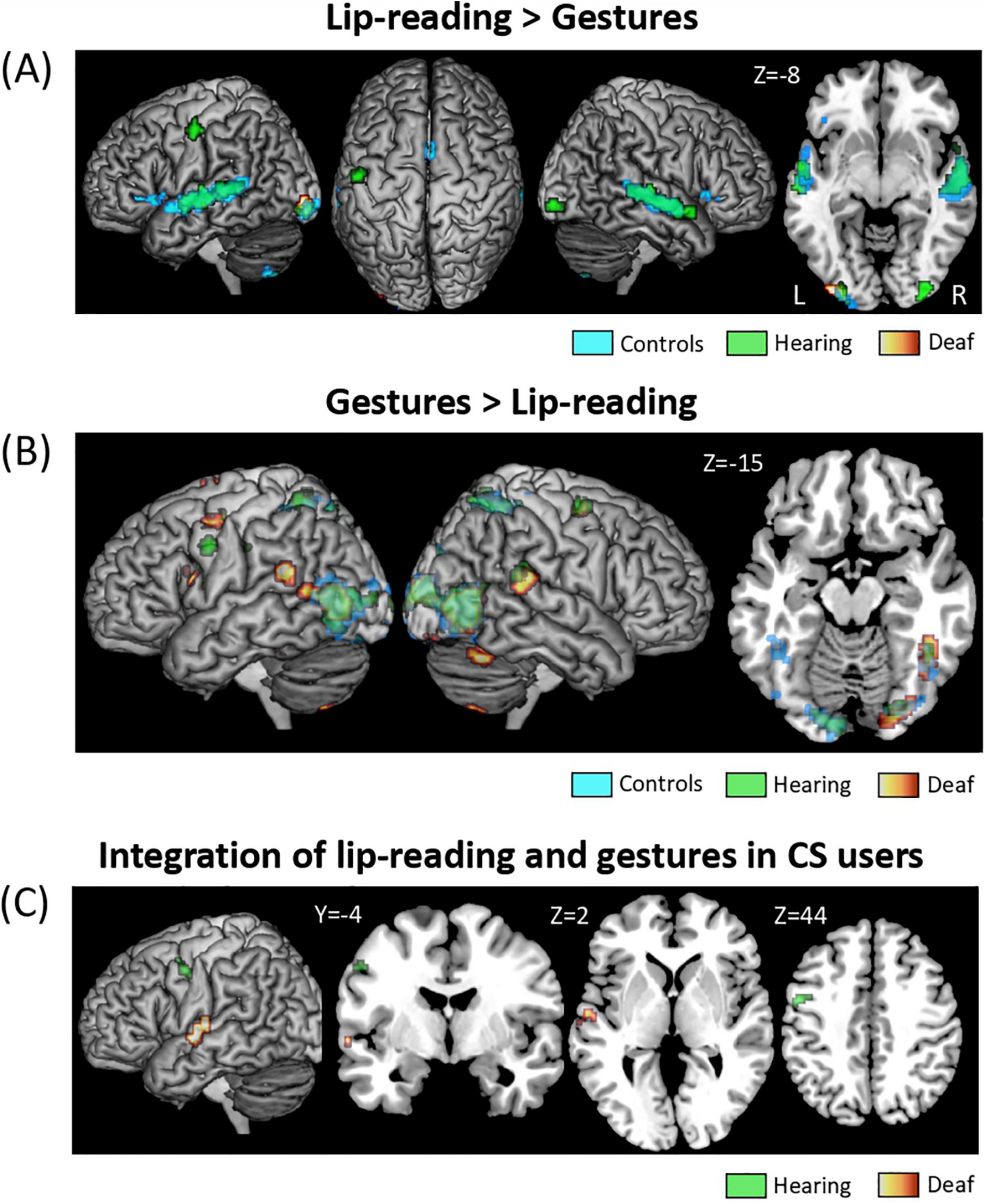
Comparison of activations by lip-reading and Gestures. Activations in Hearing, in Deaf and in Control participants. (A) Activation by Lip-reading > Gestures; (B) Activation by Gestures > Lip-reading; (C) Regions integrating lip-reading and gestures in Deaf and Hearing users, as defined by the conjunction of Sentences > Lip-Reading and Sentences > Gestures (both masked by Sentences > baseline in the corresponding group, thresholded at p < 0.01 voxel-wise).

In all three groups, the Lip-reading > Gestures activated a left inferior lateral occipital region, plus the symmetrical right-hemispheric region in hearing participants (Fig. 4A). This activation was stronger in deaf participants than in controls.

Conversely, in all three groups, most sectors of the bilateral visual cortex were activated more strongly by Gestures than by Lip-reading, including the occipital poles, inferior and lateral occipital cortex, and fusiform region (Fig. 4B). This superiority of Gestures over Lip-reading was stronger in controls than in deaf participants (masked by Gestures > baseline in Controls) in the left lateral occipital cortex, the same region found before with the equivalent Lip-reading > Gestures contrast.

###### 3.2.2.3.2 Language areas

Considering our second question, the previous analyses showed that language areas were activated by lip-reading in all groups, but by Gestures in CS users only. This resulted in the activation of those areas by Lip-reading > Gestures (Fig. 4A) only in the hearing and control groups: in the bilateral STG/STS in both groups, plus the left precentral gyrus in hearing users, and the bilateral IFG and SMA in controls. Accordingly, the deaf group showed weaker activation than controls in the bilateral IFG, bilateral STS, and left pSTG/SMG, than hearing participants in the left precentral and postcentral gyri, and than both the control and hearing groups in the bilateral SMA.

For the opposite contrast of Gestures > Lip-reading (Fig. 4B), there was no activation in language areas in controls, who had no understanding of Gestures. Conversely, deaf participants showed stronger activation to Gestures than to Lip-reading in the left IFG, right posterior middle frontal gyrus, SMA, left SMG, and bilateral pSTG. Hearing participants stood in-between the other groups, with activation restricted to the left precentral gyrus and the right pSTG. The overall stronger activation by Gestures than Lip-reading in deaf participants was obvious when comparing this contrast in Deaf > Controls and in Deaf > Hearing (masked by Gestures > baseline in Deaf): both comparisons showed almost identical activation in the left IFG, the bilateral STS, the pSTG, the SMA, and the insula. The Deaf > Hearing contrast additionally activated the left precentral gyrus.

###### 3.2.2.3.3 Dorsal attentional network

Finally, the bilateral IPS-FEF, which were activated only by Gestures in the hearing users (Supplementary Fig. 3B) and to a lesser degree in controls (Fig. 3D-E), naturally appeared in the Gestures > Lip-reading contrast in those two groups (Fig. 4B).

#### 3.2.3 Integration of lip-reading and CS gestures

At the heart of the CS system is the integration of ambiguous lip-reading and gestural cues to identify unique syllables. To address our third question and identify regions where this integration would take place, we used the “max criterion” of integration (Beauchamp, 2005;Ross et al., 2022). In each group, we looked for regions showing larger response for Sentences than for both Lip-reading and Gestures, restricting this analysis to regions activated during CS perception (voxel-wise p < 0.01). In deaf participants, this conjunction showed activation in the left mid STS (MNI -58 -11 0). In hearing users, there was activation in the left precentral gyrus (MNI -50 2 46;Fig. 4C). Lowering the voxel-wise threshold to p < 0.01 showed additional activations in hearing users in the left IFG, and in the left SMA and anterior cingulate gyrus. Controls did not show any integrating region. Direct comparison between the two groups confirmed a superiority of the deaf and the hearing groups in the left superior temporal and precentral regions, respectively, although with limited statistical strength (seeSupplementary Results 2).

#### 3.2.4 Individual activation in category-selective occipitotemporal regions

In the introduction, we put forward specific predictions concerning the activation of the VWFA, the FFA, and the EBA. In order to identify those regions based on their preferences for categories of visual stimuli, we presented pictures of written Words, Faces, Bodies, Tools, and Houses. A full presentation of this experiment is included in theSupplementary Results 1. We contrasted each category minus the average of all the others. This showed the usual mosaic of occipitotemporal category-specific regions (Supplementary Table 8), with no difference between the groups in these regions. Using the conjunction of the three groups, the bilateral FFA (42 -51 -20 and -42 -56 -22) and EBA (50 -71 6 and -48 -78 6) overlapped, respectively, with the fusiform and lateral occipital activations induced by the perception of cued-speech stimuli (Supplementary Results 1). Importantly, the VWFA, lateral to the FFA, was not activated by the perception of Sentences, Lip-reading, nor Gestures.

**Fig. 5.**
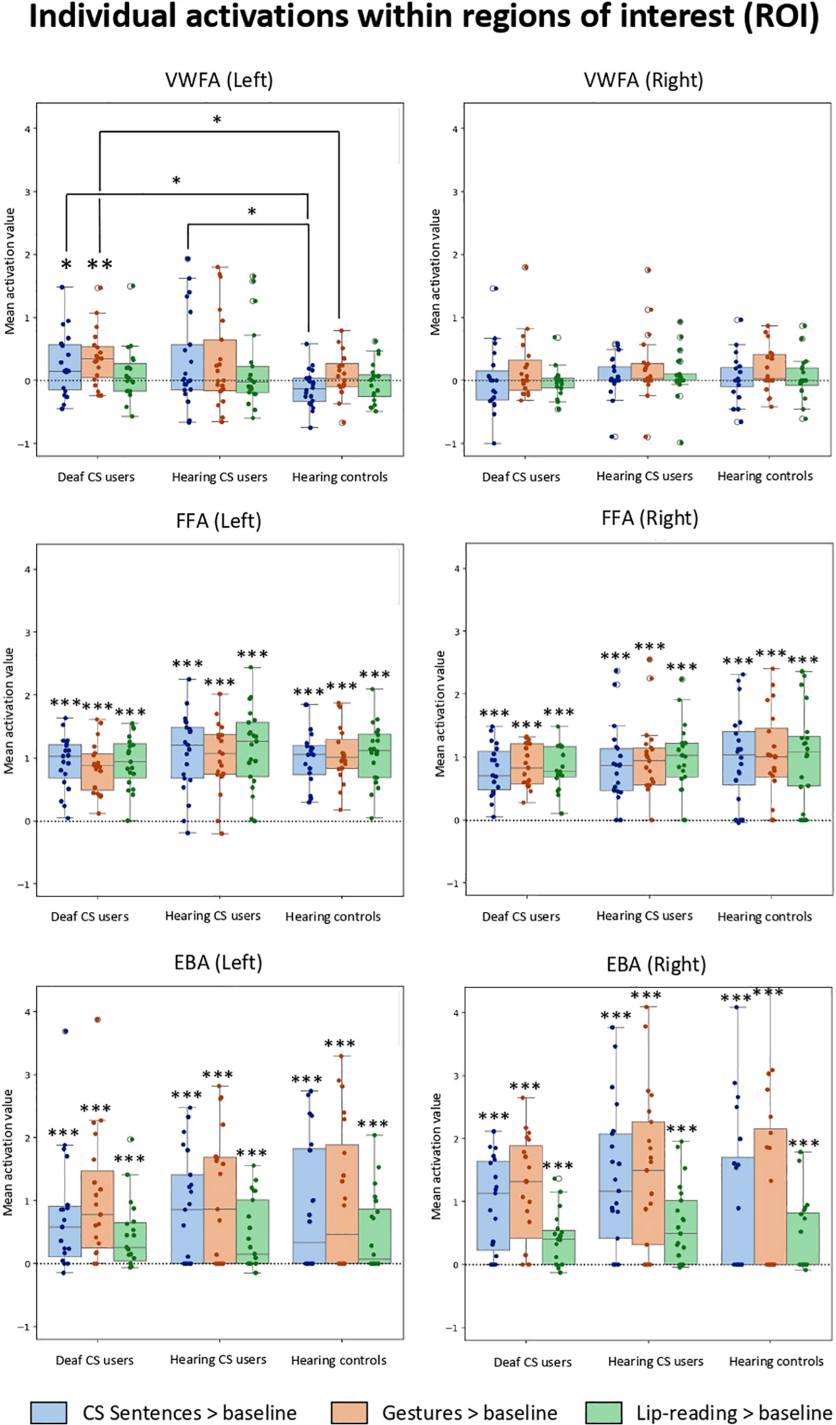
Individual activations within regions of interest (ROI). Individual activations of CS Sentences > baseline, Gestures > baseline, and Lip-reading > baseline within spherical regions of interest (ROI) centered on individual peaks.

To go beyond group-level analyses, we created spherical regions of interest (ROI) centered on the individual peaks of the VWFA and its right counterpart, and the bilateral FFA and EBA. We computed individual activations by contrasts from the main experiment, within those ROIs (seeSection 2;Fig. 5). We performed Bayesian one-sample t-tests to assess the existence of activation by CS Sentences, Gestures, and Lip-reading relative to baseline.

In deaf CS users, there was strong evidence of activation of the left VWFA by isolated Gestures (BF_10_= 28.4, effect size: Cohen’s d = 1.15), weak evidence of activation by full Sentences (BF_10_= 2.1, d = 0.01), and weak evidence for*the absence*of activation by Lip-reading (BF_10_= 0.52, d = 0.37). In the two other groups and in the right counterpart of the VWFA, Bayes factor ranged from BF_10_= 0.33 to BF_10_= 1.87, that is from a weak support for the absence of activation to a negligible support for the existence of activation (Supplementary Table 5). Accordingly, pairwise group comparisons conducted in each condition showed that deaf CS users activated more the VWFA than controls when viewing Sentences (BF_10_= 7.27, d = 0.94) and when viewing isolated Gestures (BF_10_= 2.03, d = 0.71). Hearing CS users also activated more the VWFA than controls when viewing Sentences (BF_10_= 3.55, 0.78). Other group comparisons showed no significant results (all BF_10_< 1).

In contrast, activation was highly significant in the individual FFA in all groups x conditions, both in the left and right hemispheres (all BF_10_> 10^4^). The same was true for the bilateral EBA (all BF_10_> 10). Pairwise group comparisons conducted in these ROIs showed no significant result (all BF_10_< 1). In summary, evidence supported activation of the VWFA only when deaf participants were watching CS Gestures, and predominantly when Gestures were isolated.

#### 3.2.5 Activation by audible sentences

As predicted, the comparison of Audible > Silent Sentences (masked by Audible Sentences > baseline) showed no activation in deaf participants, while the conjunction of control and hearing participants (masked by the conjunction Audible > baseline of the same groups) showed massive activations in the bilateral middle temporal gyrus (MTG) and STG (Supplementary Fig. 4A;Supplementary Table 6). The controls and hearing groups did not differ.

#### 3.2.6 Activation by pseudo-sentences

Finally, we compared activations elicited by silent meaningful Sentences and by Pseudo-sentences to delineate the subset of regions activated during semantic processing. We first contrasted the silent meaningful Sentences > Pseudo-sentences (masked by silent meaningful Sentences > baseline). As expected, controls did not show any activation. More unexpected, deaf participants only showed a small activation cluster in the left lingual gyrus, while hearing participants showed activation in the left IFG and the bilateral insula (Supplementary Fig. 4B;Supplementary Table 7). Pairwise comparisons between groups showed no significant differences.

The paucity of activations induced by this contrast is consistent with real words generating little more activation than pseudo-words (Binder et al., 2009;Taylor et al., 2013). This is because the processing of real and pseudo-language involves the same brain systems, and that pseudo-words may require additional effort than real words. However, we will later derive valuable insights into individual variability among CS users from this contrast (see alsoSupplementary Results 3).

## 4 Discussion

In the introduction, we put forward a series of four questions or predictions, which we will now discuss in turn: Specialization in the visual cortex, activation of core language areas, integration of CS components, and specificities of CS processing in hearing users.

### 4.1 Visual perception of cued speech

#### 4.1.1 The role of the VWFA

Cued speech sentences activated the visual cortex almost identically across all groups, including the posterior, inferior, and lateral occipital cortex, and the fusiform region. The two latter regions overlapped with the EBA and the FFA, respectively, which is unsurprising considering that videos always featured a facing and gesturing human person (Supplementary Results 1). In contrast, activation did*not*extend to the VWFA, particularly in the deaf participants, consistent with previous findings (Aparicio et al., 2017).

However, closer scrutiny based on individual ROIs and Bayesian statistics revealed a more interesting pattern. While the VWFA was never activated in the Hearing and Control groups, there was strong evidence that Gestures activated the VWFA of deaf participants, particularly when presented in isolation. This is a decisive finding in the debate on cortical specialization in the VOTC. According to the bottom-up theory, the VWFA site becomes specialized for letter strings due to its preference for their visual features (Srihasam et al., 2014). The opposing top-down theory claims that the reading-specific properties of the VWFA result entirely from its predisposition to act as an interface between object-oriented vision and language (Price & Devlin, 2011). The current data suggest a contribution of both approaches. A purely bottom-up theory would predict that, irrespective of CS knowledge, the VWFA should be activated neither by lip-reading nor by gestures, which have no resemblance to letters. Conversely a pure top-down theory would predict that both types of cues should activate the VWFA, as they both convey visual information with a phonological value. Evidence of a top-down contribution is best illustrated by the activation of the VWFA of competent readers by auditory speech (Cohen et al., 2021;Dehaene et al., 2010), by fingerspelling (Emmorey et al., 2015), but also by tactile Braille letters (Amedi, 2002) or by auditorily coded letter shapes (Reich et al., 2011). A fortiori, deaf participants, who have a fluent comprehension of cued speech, may perceive hand configurations as shapes conveying a phonological content comparable with the content of alphabetic or syllabic scripts.

Hand configurations would thus be processed along the same pathways as conventional alphabets, including the VWFA. When perceived in isolation, gestures are the unique source of comprehension, and the VWFA activation is, therefore, intense. During the perception of full CS sentences, lip-reading plays a predominant role, gestures are less necessary, and the VWFA is weaker. As to the VWFA of hearing CS users, whose automatic CS comprehension was poor, and of control participants, it was not attuned to the automatic recognition of hand configurations, and was, therefore, not activated. Finally, it is noteworthy that although lip-reading also carries phonological information, it did not activate the VWFA. Contrary to letters and CS gestures, lip-reading is not an arbitrary symbolic code processed by recycling the ventral visual cortex (Dehaene & Cohen, 2007), but the visible aspect of speech, analyzed through universal mechanisms involving interconnected occipital and superior temporal regions (Peelle et al., 2022). Even assuming that the VWFA contributes to interfacing vision and language in CS experts, it cannot ensure the whole processing of hand gestures which, as indicated in the introduction, is largely supported by the lateral occipital cortex (Bracci et al., 2018;Wurm & Caramazza, 2019).

#### 4.1.2 The role of the LOTC

During reading, the identification of letters must be invariant for irrelevant visual changes. There is indeed evidence that the VWFA shows invariance for case, font, and position (Cohen & Dehaene, 2004). Similarly, the identification of gestures during CS perception should be invariant for the identity of the coder, the viewpoint, the position of the display in the visual field, etc. Parts of the bilateral LOTC show preference for hands or of bodies (Bracci et al., 2010) and, using multivariate pattern analysis (MVPA),Bracci et al. (2018)found that the hand-selective regions encode hand postures in a viewpoint-invariant manner. Moreover, the LOTC cortex is also sensitive to more abstract features of gestures, such as whether they involve social interactions, and whether they involve object manipulation (Lingnau & Downing, 2015;Wurm et al., 2017;Wurm & Caramazza, 2019). Sociality is a relevant feature of CS gestures, due to their intrinsic communicative function. Accordingly, we found between-groups differences in the LOTC, likely resulting from the tuning of the visual cortex to the expert processing of CS (seeFigs. 2Band3E).Aparicio et al. (2017)also found higher activation of the left LOTC during CS perception in deaf users than during a still control condition. Thus, although the current design does not allow us to fully probe the functional properties of the left LOTC, this region likely implements the identification of CS gestures.

#### 4.1.3 Fingerspelling and sign language

Although it is not the place to review the whole evidence on sign language perception, one may wonder, considering the recycling of the LOTC for CS deciphering, and of the VWFA for reading, whether those regions are also involved in the other two systems that use hand gestures as a linguistic communication channel: sign language and its ancillary alphabetic fingerspelling. The three systems differ deeply in the function of gestures: In sign language, gestures refer to abstract linguistic entities such as morphemes, irrespective of the sound or orthographic content of their translations in spoken languages, while they denote candidate syllables in CS, and letters in fingerspelling.

The bilateral LOTC being involved in all manners of gesture perception (Yang et al., 2015), it is activated by both fingerspelling and sign language, in deaf signers and naïve controls (Emmorey et al., 2015;Liu et al., 2017;MacSweeney, 2002;Waters et al., 2007). Moreover, although the precise overlap of activations is difficult to ascertain across studies, activation by fingerspelling was stronger than by sign language in the inferior part of the bilateral LOTC, overlapping with the LOTC region where we found sensitivity to CS knowledge, while sign language activated preferentially more dorsal LOTC regions (Emmorey et al., 2015). The VWFA also was activated by fingerspelling more than by sign language, in deaf signers only. On this basis, one may speculate (i) that ventral LOTC activation reflects the expertise in CS and fingerspelling perception, which both rely on the expert identification of static hand configurations with a language-related content and (ii) that the VWFA is specifically attuned to the identification of letters, both printed and fingerspelled, as it is also engaged in reading novel letters shaped like faces or houses (Martin et al., 2019;Moore et al., 2014). Those hypotheses should be further assessed in appropriate within-subject studies.

#### 4.1.4 Lip-reading

While gestures activated most of the visual cortex more strongly than lip-reading, a patch of left posterior lateral occipital cortex showed the opposite preference in all groups. This region is posterior to the face-selective FFA and OFA, and may provide input to later stages of face processing (Elbich et al., 2019). Importantly, and contrary to gestures, we found no difference between groups in the activation by lip-reading stimuli in any part of the visual cortex. This negative finding is in keeping with the fact that this communication channel is operational in all participants. Indeed, the activation of most language areas by pure lip-reading stimuli does not differ across groups.

### 4.2 Access to language areas

#### 4.2.1 Commonalities across groups

With variation across groups and conditions, CS consistently activated core language areas, a set of lateral frontal and temporal regions (with more extended activations in the left hemisphere;Fedorenko et al., 2024). Silent CS sentences elicited a set of common activations across the three groups, including controls ignorant of the CS code, in the inferior frontal, precentral, and STS/STG regions (with more extended activations in the left hemisphere;Fig. 2A). Such commonalities resulted from the shared use of lip-reading as an input code to language. Accordingly, a similar pattern of commonality was induced by pure lip-reading stimuli (Fig. 3A), but not by pure Gestures (Fig. 3C). Indeed, pure Gestures induced extensive activation of language areas in deaf participants only (Fig. 3D-E), which further confirms that hearing users of CS, who mastered CS less fluently than deaf participants, had a brain activation pattern in most respects comparable with the one of controls.

#### 4.2.2 Cued-speech expertise

Within this set of common regions, activation by CS sentences was stronger in deaf participants than in both controls (bilateral pSTS/STG and left IFG;Fig. 2B), and hearing CS users (left pSTS/STG;Fig. 2C). This profile nicely parallels the superior performance of deaf participants in CS comprehension as compared with naïve controls, but also, to a lesser degree, to hearing CS users (Fig. 1B).

The fact that the perception of isolated gestures is sufficient to trigger linguistic activations in Deaf users may come as a surprise, as this CS component is by itself very ambiguous (approximately nine possible syllables for each hand shape x position combination). Interestingly, 14 of the deaf participants declared during the debriefing have the impression of understanding at least partially these stimuli. Anecdotal testimonies exist on very proficient CS users being able to communicate using only the gestural part of CS (e.g.Weill, 2011), for example when they are talking at a distance or with their mouth full while eating. Such communications often happen in familiar contexts, so that top-down pragmatic influences certainly play an important role. There are also purely lexical constraints of the interpretation of gestures. Sequences of gestures deprived of lip-reading cues have huge numbers of potential phonological interpretations. As each gesture corresponds to ~9 syllables, any sequence of 3 gestures may receive ~9*9*9 = 729 phonological interpretations. Only a small minority of those interpretations correspond to real words, which could strongly facilitate comprehension (Ferrand et al., 2018). Still the degree of ambiguity in this linguistic input remains very high and linguistic top-down inferences seem at first insufficient for such decoding, even in a deaf population with a daily practice. Future experiments on the contribution of those factors to such performances would improve our understanding of CS processing and of the role of top-down inferences in language comprehension.

#### 4.2.3 Language and perception

The bilateral posterior tip of the STG and precentral cortex shared an intriguing pattern of activation. They were activated by CS sentences in all groups (Fig. 2A), which would be consistent with their participation in the core language system, as discussed before. Moreover, the pSTG was more activated in deaf participants than in the other groups by Sentences (Fig. 2B-C), by Lip-reading (Fig. 3B), and by Gestures (Fig. 3D-E), suggesting that it contributed to CS expertise. However, those regions were also activated by Gestures across all groups, a surprising finding considering that Gestures carried no linguistic meaning for controls (Fig. 3C-D). One possible account of this pattern is that, beyond language, both regions are also involved in action perception, in addition to the visual cortices discussed before (Wurm & Caramazza, 2022). Indeed, meta-analyses show reproducible activation of the bilateral precentral gyrus and the pSTG during action observation, an activation that may even be stronger than during actual action execution (Caspers et al., 2010;Hardwick et al., 2018). Moreover, strokes affecting either of those regions impair the identification of biological motion more than control moving stimuli (Saygin, 2007).

Hence, one may propose that the precentral gyrus and the pSTG act as an interface between the visual analysis of CS components and their integration in language comprehension. The same idea may apply to other visual communication systems, and indeed the left pSTG is also strongly activated in deaf signers by sign language and by fingerspelling, as compared with various controls (Emmorey et al., 2015;MacSweeney, 2002;Waters et al., 2007). More generally, this hypothesis fits well with the general role of the pSTG and the adjacent SMG whenever phonology has to be interfaced with orthography, sensorimotor processing, or lip-reading (Binder, 2017;DeWitt & Rauschecker, 2012;Hickok & Poeppel, 2016;Hickok et al., 2018;A. Martin et al., 2016).

### 4.3 Integration of lip-reading and CS gestures

In this study, we propose that lip-reading and gestural information are integrated into CS information in the left mid STS in deaf users, and in the motor regions, mainly the left precentral gyrus, in hearing users (Fig. 4C). In deaf participants, the STS location suggests that integration first occurs at the phonological level, with lip-reading and gestures information converging to form a unified phonological representation of the two components. This result runs against the alternative hypothesis that integration would occur in the visual cortex, with syllables represented as complex visual combinations of gestures and mouth configurations.

In hearing users, we found evidence of integration in motor areas, suggesting that they used a different strategy than deaf participants. Rather than relying on a unified phonological information, they relied on their good CS production skills to integrate the lips and hand movements, a process which naive controls were not able to perform. This result strengthens the idea of a precentral gyrus interfacing the visual analysis of CS components and their integration in language comprehension in expert CS producers, while the activation in controls may merely reflect the usual role of the precentral cortex in action observation. This difference in integration regions between deaf and hearing users suggests that the groups did not only differ in their proficiency in CS comprehension, but also in the very strategy which they used to process CS.

Behavioral studies indirectly suggested that CS perception may also rely on executive functions, such that each syllable would be explicitly deduced from the perceived CS components, successively taking into account gestures to constrain the interpretation of the subsequent lip movements (Attina et al., 2005). In the absence of any integration in prefrontal regions, we found no evidence to support this hypothesis (for a review seePeiffer-Smadja & Cohen, 2019).

Finally,Aparicio et al. (2017)proposed that integration occurs in the left lateral occipital cortex, based on stronger activation by full CS words than by the average of gestures and lip-reading. However, like in the present study, this region was strongly activated by full CS and by isolated gestures, and not activated by lip-reading. This pattern explains naturally the effect observed byAparicio et al. (2017), without resorting to an integration hypothesis.

### 4.4 The dorsal attentional network

Whenever the gestural component of CS was presented (Figs. 2C,3E,4BandSupplementary Results 3), we observed activation in the bilateral FEF and IPS. This activation was intense only in hearing CS users, moderate in controls, and absent in deaf participants. Those regions are involved in the tightly linked functions of spatial attention and saccadic eye movements (Pouget, 2015). As we did not record eye movements during scanning, it is possible that differences in FEF/IPS activation were associated with differences in gaze behavior. However, our recent recordings of gaze during CS perception using real size stimuli (Sarré & Cohen, 2025) did not show a different pattern in hearing users, making this possibility unlikely. More generally, frontal and parietal cortices are a source for generating attention-related signals which modulate the visual cortex from top-down (Fiebelkorn & Kastner, 2020;Mengotti et al., 2020). Among those areas, the bilateral FEF and IPS form the core of the Dorsal Attentional Network (DAN), which supports the allocation of spatial attention at locations relevant to the ongoing goals and tasks (Corbetta & Shulman, 2002). It activates for instance more during demanding than easy visual tasks, such as detecting an image in visual noise (Aben et al., 2020). Presumably, the DAN does not need to activate in the deaf participants, who are the most expert users of CS, for whom gesture identification is so automatized that it requires only minimal attentional effort. To the opposite, the hearing users of CS, who have a better experience of CS production than comprehension, require a strong attentional focus to effectively decipher the CS code. Control participants, for whom the task was too hard for additional attention to make a difference, fell in between the two other groups.

### 4.5 Open questions

We have delineated a number of mostly left-hemisphere brain regions involved in CS perception: Visual cortices where hand gestures and mouth configurations are identified, fronto-parietal regions that control visual attention in hearing CS users, distinct regions that support the integration of lip-reading and gestures depending on expertise, and core language areas where information converges. In addition, we identified correlates of individual variability among hearing CS users who differ in their CS comprehension abilities.

The current conclusions should largely apply to all language-specific versions of CS (International Academy Supporting Adaptations of Cued Speech (AISAC), 2020), which are all based on the exact same principles. Still, it would be worth studying the specific mechanisms of other CS systems, such as those using a movement component to carry the phonological tonal information.

These findings open up a wide range of questions for future research. For example, multivariate methods would allow more direct investigation of the codes supported by each brain region, for example, based on the decoding of gestures, mouth configurations, syllables, and their integration. Using time-resolved techniques (EEG, MEG), we could also extend this approach to the time domain, revealing the time course of CS decoding, integration, and comprehension. Beyond CS comprehension, the whole field of CS production, including the interface with language and the planning of motor commands, remains largely unexplored. Finally, CS is a valuable tool in the education of deaf children, and understanding the processes of CS learning may prove to be both fundamental and practical.

## Supplementary Material

Supplementary Material

## Data Availability

Group-level MRI data supporting the findings of this study are available athttps://doi.org/10.5281/zenodo.15516223. More extensive sharing would require a formal data sharing agreement. The stimulation, preprocessing, and analysis scripts are available athttps://github.com/AnnahitaSarre/CUSPEX_seeing_speech.
