# Supplementary material for: Seeing speech: Neural mechanisms of cued speech perception in prelingually deaf and hearing users
Source: Imaging Neurosci (Camb). 2025 Jun 24;3:IMAG.a.53. doi: 10.1162/IMAG.a.53 (PMC12319768; doi:10.1162/IMAG.a.53)
Supplement: Supplementary Material [file imag.a.53_supp.pdf]

Supplementary materials

Supplementary results 1 – Functional localizer: Brain activation during visual objects perception 2

Methods 2

Behavioral results 2

fMRI results 2

Comments 3

Supplementary results 2 – Integration of lip-reading and CS gestures 5

Supplementary results 3 – Individual variability among hearing participants 6

Results 6

Comments 7

References 10

Supplementary figures 12

Supplementary tables 13

Supplementary results 1 – Functional localizer: Brain activation during visual objects perception

In order to identify category-specific regions in the visual cortex, we presented pictures of written Words, Faces, Bodies, Tools, and Houses.

Methods

Static images were presented to participants, distributed among 5 visual object categories, each represented by 20 pictures: faces, bodies, French words, houses and tools (for a full description of stimuli see Zhan et al., 2023).

The experiment consisted of 12 blocks for each category of stimuli, arranged in a random order. In each block, all 20 pictures from the considered category were presented, each time in a different random order. Each picture was presented for 100 ms, followed by a 200 ms blank screen with a fixation cross. Blocks had thus a duration of 6 s, and were separated with 6 s of fixation baseline, for a total session duration of 12 minutes.

Stimuli presentation was done using Psychtoolbox Version 3 (Brainard, 1997) in MATLAB R2019b.

A white fixation cross was present at the center of the screen throughout the experiment. Participants were asked to fixate the cross, to detect the picture of a star which replaced a picture in half the blocks of each category, and to press a response button as rapidly and as accurately as possible.

Behavioral results

Participants performed close to ceiling in the outlier detection task, with a mean hit rate of 97 ± 0.04% and a mean dprime of 4.48 ± 0.43. No significant difference in mean dprime was found between groups (all Mann-Whitney U tests $p > 0.05$). The mean and variance of response times did not differ between groups (assessed respectively with Mann-Whitney U tests, all $p > 0.05$, and Levene tests, all $p > 0.05$).

fMRI results

Category-specific activations.

We contrasted each category minus the average of all the others, computing the conjunction of those contrasts across the three groups. This showed the usual mosaic of occipitotemporal category-specific regions (Section A of below figure; Suppl table 8). Words activated the VWFA in the left occipitotemporal sulcus, the left pSTS/STG and IFG, plus the bilateral calcarine region. Faces activated the fusiform gyrus including the right FFA, the right pSTS/STG, plus the bilateral calcarine sulcus and the right cuneus. Bodies activated the Extrastriate Body Area (EBA) in the bilateral LOTC. Tools activated the bilateral collateral sulcus, the LOTC (ventral and anterior to the EBA), the right middle and superior occipital gyri, plus the bilateral IPS. Finally houses activated the PPA in the bilateral collateral sulcus and lingual gyrus, plus the right precuneus and the occipital poles.

Pairwise comparisons of category-specific activations between groups only showed increased activation in the right pSTG by Faces, in deaf participants relative to controls (masked by the same contrast in Deaf) (Section A of below figure).

Activation relative to baseline.

Beyond category-specific activation of the VOTC, pictures activated broader cortical areas relative to baseline. Pairwise comparisons showed that the control and hearing groups did not differ. The comparison of the Deaf group minus the average of the hearing and control groups (masked by activation in Deaf participants) for each visual category relative to baseline showed that deaf participants activated the right pSTS/STG more than the other participants in all categories but Houses. This activation extended more anteriorly for Words and Faces. Additional activations were present in the left collateral sulcus when viewing Faces and Bodies, and in the left pSTS/STG when viewing Faces (more posterior and smaller than in the right hemisphere) (Section C of below figure; Supple table 9).

In summary, we found in all groups the usual category-selective activations in the occipitotemporal cortex. Group differences were largely restricted to the right pSTS/STG, which was more activated in deaf than in hearing and control participants for most categories of pictures. This over-activation in the Deaf group was more marked for Faces, resulting in a face-specific activation.

Comments

Activation of the right pSTS in the deaf population. Conducting a typical mapping of category-specific visual areas, we found activation for faces in the right pSTS in deaf participants compared to the two hearing groups (Section A of below figure). This over-activation has been observed in early deaf signers (Benetti et al., 2017) as well as in the context of late deafness, and it diminishes after successful cochlear implantation (Lazard & Giraud, 2017; Rouger et al., 2011). It may reflect a reassignment of the so-called “temporal voice-selective area” (Belin et al., 2000), selective for human voices in the hearing population and for face processing in deaf individuals. Assessing non-specific activations, we found that, even though the pSTS has a preference for faces, other visual categories, i.e. words, bodies and tools, elicited an over-activation in deaf participants compared to the two hearing groups (Section C of below figure). Hence those findings may not be only linked to the region’s position in the secondary auditory cortex, but also to a general sensitivity to visual inputs. In both our study and Aparicio et al. (2017), this region was activated during CS perception in all three groups, with over-activation in deaf participants compared to controls and hearing users. A general increase of activation in this region in the deaf population for such visual input aligns with this finding.

Activation of the VWFA in the deaf population. The lack of any group difference during word reading in this experiment is in agreement with the literature. As reviewed by Hirshorn et al. (2022), VWFA activation during reading is largely similar in deaf and in hearing persons, apart from subtle differences in the influence of phonological skills (Emmorey et al., 2016; Glezer et al., 2018).

Activation of the left collateral sulcus in the deaf population. Static pictures of faces and bodies induced stronger activation in the left collateral sulcus of deaf than of hearing participants (Section C of below figure). The topography of this activation cluster overlaps with area V4v (Eickhoff et al., 2005; Rottschy et al., 2007). This richly connected region is thought to implement an early stage of object perception, and to guide perceptual decisions (for a review see Pasupathy et al., 2020). In deaf CS experts, for whom visual cues are the exclusive source of language perception, there is thus an increase in V4 sensitivity, specific to the two classes of objects which convey linguistic signals: human faces and bodies. Interestingly, this activation of area V4v also showed negative correlation between CS proficiency and activation by Gestures (Supplementary results 3). Despite the main experiment and

the functional localizer using very different stimuli and tasks, this overlap further supports the above hypothesis that the left V4 plays a role in early perceptual stages of expert CS perception.

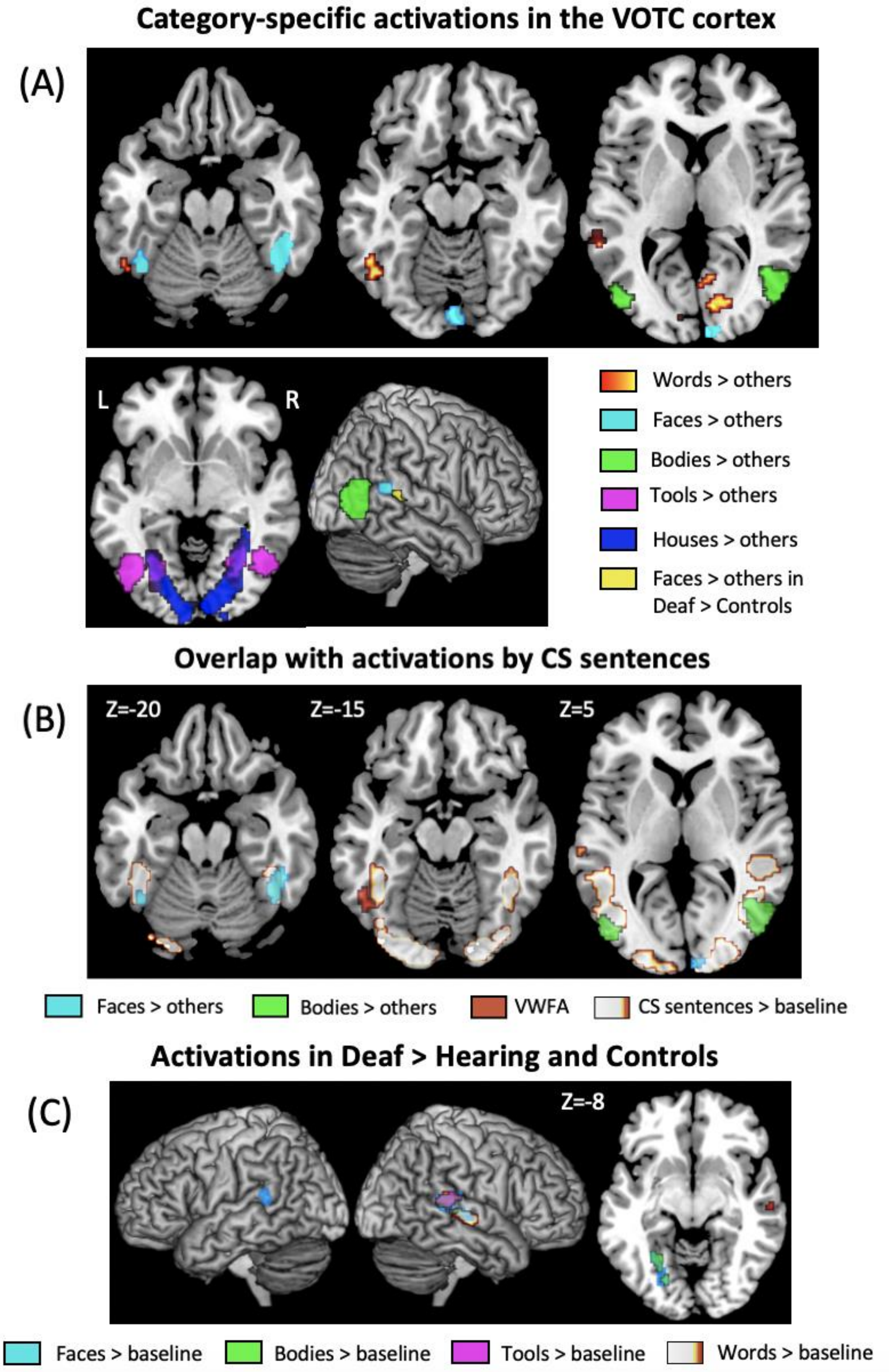

(A) Activations by each category of visual objects > all other categories, common to the three groups. The only difference between groups was a higher selective activation by faces in Deaf than in Control participants; (B) Overlap of the category-selective activation for faces (FFA) and bodies (EBA), with the fusiform and lateral occipital activation by CS; (C) Larger activation relative to baseline in deaf than in the average of hearing and control participants

Supplementary results 2 – Integration of lip-reading and CS gestures

As described in the main text, when looking for regions involved in the integration of gestures and lip-reading cues, we applied the "max criterion", according to which one signature of integration is the conjunction of a higher activation for full CS sentences than for both gestures and lip-reading alone. We found distinct regions in the two groups of CS users in which this criterion was significantly met: a left superior temporal region in the deaf group and a left precentral region in the hearing group. Based on the way conjunction statistics were computed (Friston et al., 2005; Nichols et al., 2005), it was not possible to directly compare the conjunction of contrasts between the two groups using the same statistical tool. We therefore compared groups using a slightly different method, showing group differences reaching an excellent topographical match with the result obtained in the separate groups.

In each participant, we computed the overlap of activations by Sentences > Gestures and Sentences > Lip-reading (each with a threshold of $p < 0.001$ voxelwise), thus identifying the voxels satisfying the "max criterion". Individual overlap maps were binarized and smoothed (FWHM 8 mm), and compared between groups with non-parametric t-tests using the SnPM toolbox (Nichols & Holmes, 2002). At a relatively low statistical threshold (voxelwise $p < 0.01$), within the regions activated by full sentences, we found group differences in the same regions already identified in separate groups. There was larger integration in the deaf than in the hearing group only in the left superior temporal cortex. Conversely, larger integration in the hearing than in the deaf group was present in a small left precentral cluster.

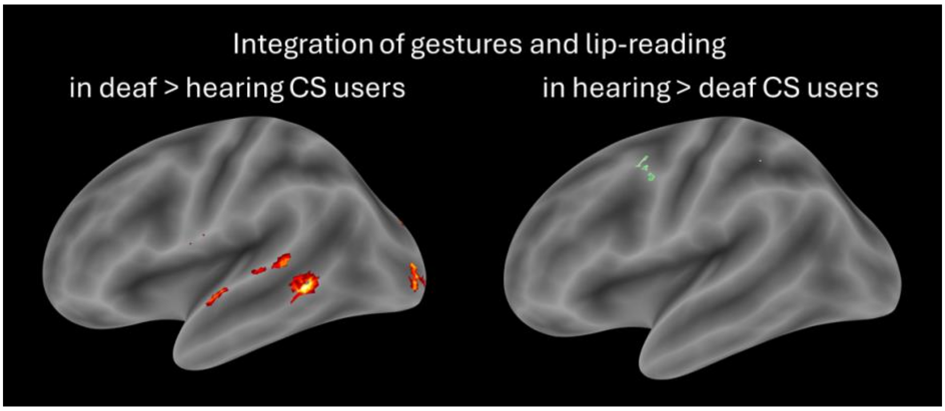

Regions with different integration in the two groups, as measured by the conjunction of the contrasts of Sentences > Gestures and of Sentences > Lip-reading. Left panel: left temporal regions more involved in integration in the deaf group (hot). Right panel: left precentral regions more involved in integration in the hearing group (green) (voxelwise $p < 0.01$).

Supplementary results 3 – Individual variability among hearing participants

CS mastery was variable across hearing users, while the deaf and control groups were quite homogeneous (Figure 1B). Looking for the imaging correlates of such behavioral variability, we studied the correlation of the main contrasts of interest (Sentences, Lip-reading, and Gestures > baseline, and Sentences > Pseudo-sentences, with no masking) with two individual behavioral measures: the proficiency at understanding CS sentences (as measured during the pretest), and the age of CS learning (see figure 2 below for individual activation plots).

Results

First considering the contrast of Sentences > baseline, we found a negative correlation with the participants' age of CS learning, in the left middle cingulate (with a more extended activation in the left hemisphere) and superior medial frontal gyri (Figure 1A below). Furthermore, there was a negative correlation with sentence comprehension in the left middle occipital gyrus (Figure 1B below). This overall effect was present separately for the comprehension of "Intermediate" and "Difficult" sentences (for the latter, activation extended to the left lingual gyrus), but not of "Easy" sentences, even when lowering the voxelwise threshold to $p < 0.01$.

We then examined the contrast of Lip-reading > baseline. As for Sentences > baseline, there was a negative correlation with age of CS learning in the bilateral cingulate and medial superior frontal gyri, as well as in the right middle STS, the left angular gyrus and the bilateral superior and middle frontal gyri (Figure 1A below). There was no correlation with sentence comprehension score.

Turning to the Gestures > baseline contrast (Figure 1B below), we found, similarly to Sentences > baseline, negative correlation with sentence comprehension in the left middle occipital gyrus and the left lingual gyrus (still absent with "Easy" sentences). Moreover, there was a positive correlation with sentence comprehension in the left angular gyrus, present with all levels of sentence difficulty (still absent with "Easy" sentences).

We then examined the contrast of meaningful Sentences > Pseudo-sentences (Figure 1A below; see also figure 2C below for illustration of the underlying mechanism), and found a positive correlation with sentence comprehension in the left IFG. The same result was present when examining "Difficult" sentences separately, while "Intermediate" positively correlated with the right superior medial frontal gyrus and the left cerebellum. No positive correlation was found with "Easy" sentences. Furthermore, there was a negative correlation with sentence comprehension in the right angular gyrus, the bilateral superior and superior medial frontal gyri, and the right posterior orbital frontal cortex. Examined separately, all levels of sentence difficulty yielded a negative correlation in some subset of these regions, plus the left anterior MTG and bilateral cingulate cortex for the "Difficult" sentences, and the right posterior MTG for the "Easy" sentences.

Most regions involved in those correlations belong to the so-called default mode network (DMN; Smallwood et al., 2021; Thomas Yeo et al., 2011), including the mesial prefrontal and parietal regions, the angular gyrus, and the right MTG. Overall, deactivation of the DMN diminishes as hearing users gain CS expertise, so that proficient users may require less externally oriented attention.

Comments

Our findings in hearing CS users may be summarized in two points. First, their behavioral mastery of CS comprehension was both substantially worse than the deaf participants', and quite variable across individuals (Figure 1B). Second, at the group level, their pattern of brain activation was remarkably similar to the control group's, from which it differed mostly by stronger activation of the dorsal attentional network (Figure 2B). In order to make sense of individual variability and to clarify whether hearing CS users follow a spectrum of activation patterns between controls and deaf CS experts, we studied the correlations across participants between activations on the one hand, and CS comprehension proficiency and CS age of acquisition on the other hand. Despite a relatively limited number of data points for correlation analyses, we could account for part of the individual variability.

Age of acquisition and the DMN. Activation by Sentences and Lip-reading was negatively correlated with the age of CS learning in regions typical of the default mode network (DMN, Smallwood et al., 2021; Yeo et al., 2011), including mesial prefrontal and parietal regions, the angular gyrus, and the right MTG (Figure 1A below). Activation in the DMN is typically suppressed whenever attention is focused on external task demands. In participants who acquired CS at an earlier age, the DMN was close to baseline, suggesting that highly automatized CS perception required little attention (Figure 2A below). The DMN was more deactivated in later learners, who required more focused attention to process the stimuli. This is congruent with the decrease in DMN deactivation after practicing perceptual categorization tasks (Shamloo & Helie, 2016), and with the smaller DMN deactivation in tasks that require a lower than a higher effort (Weber et al., 2022).

Cued speech proficiency and sentence meaning. The correlation analyses showed that the DMN was less deactivated in pseudo- compared to meaningful sentences, a result in line with previous research on the processing of spoken or written real and pseudo-words (Binder et al., 2009; Graves et al., 2017). This difference was larger in experts, as it increased with sentence comprehension scores (Figure 1C below; Figure 2C below). Note moreover that the deactivation of the left angular gyrus (AG) component of the DMN decreased as expertise increased, a further mark that in experts CS processing required less externally oriented attention (Figure 1B below).

Conversely, the left IFG was more activated in meaningful compared to pseudo sentences (Suppl figure 2B), in agreement with previous studies (Chen et al., 2023). Again, this effect of lexicality was larger in experts (Figure 2C below), as it increased with sentence comprehension scores, revealing a better distinction between meaningful and pseudo-sentences in the core language system of participants with better mastery of CS.

Cued-speech proficiency and the left occipital cortex. The activation by Sentences and by Gestures was negatively correlated with CS proficiency in left ventral and lateral occipital regions (Figure 1B below; Figure 2B below). The imaging literature on the acquisition of perceptual skills is complex, showing both increases and decreases of activation in different regions for mirror reading (e.g. Poldrack et al., 1998), or more complex inverted U-shaped curves over the period of reading acquisition (Dehaene-Lambertz et al., 2018). The current study does not allow for a decisive interpretation of this finding, which nevertheless fits with our general proposition that CS expertise rests on the functional tuning of left-hemispheric visual regions.

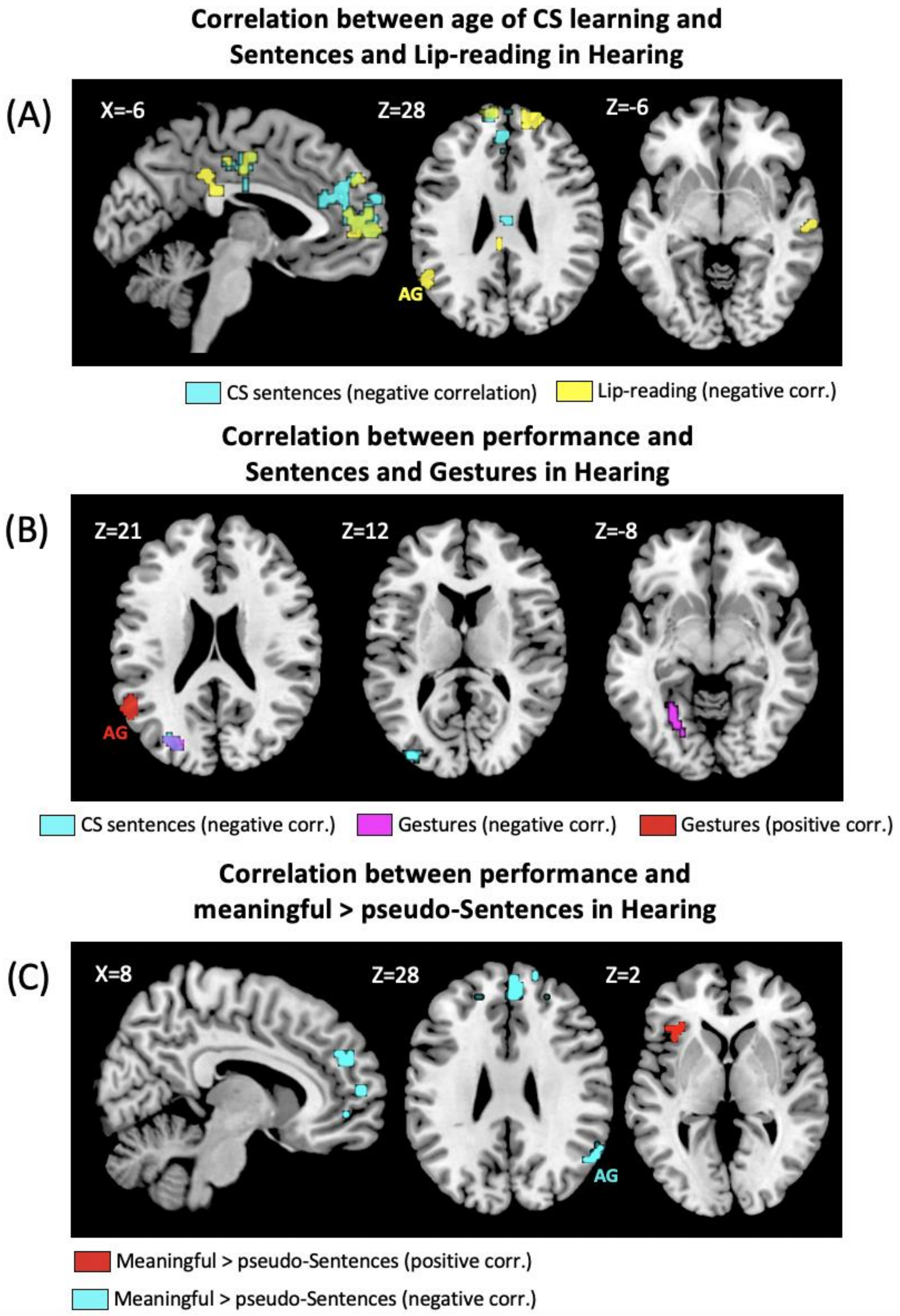

Figure 1 – Individual variability among hearing participants. (A) Negative correlation of activations by CS Sentences and by Lip-reading with age of CS learning; (B) Negative correlation of activations by CS Sentences and by Gestures with CS comprehension performance, and positive correlation of activations by Gestures with CS comprehension performance; (C) Negative and positive correlation of activations by meaningful > pseudo-Sentences with CS sentences comprehension performance.

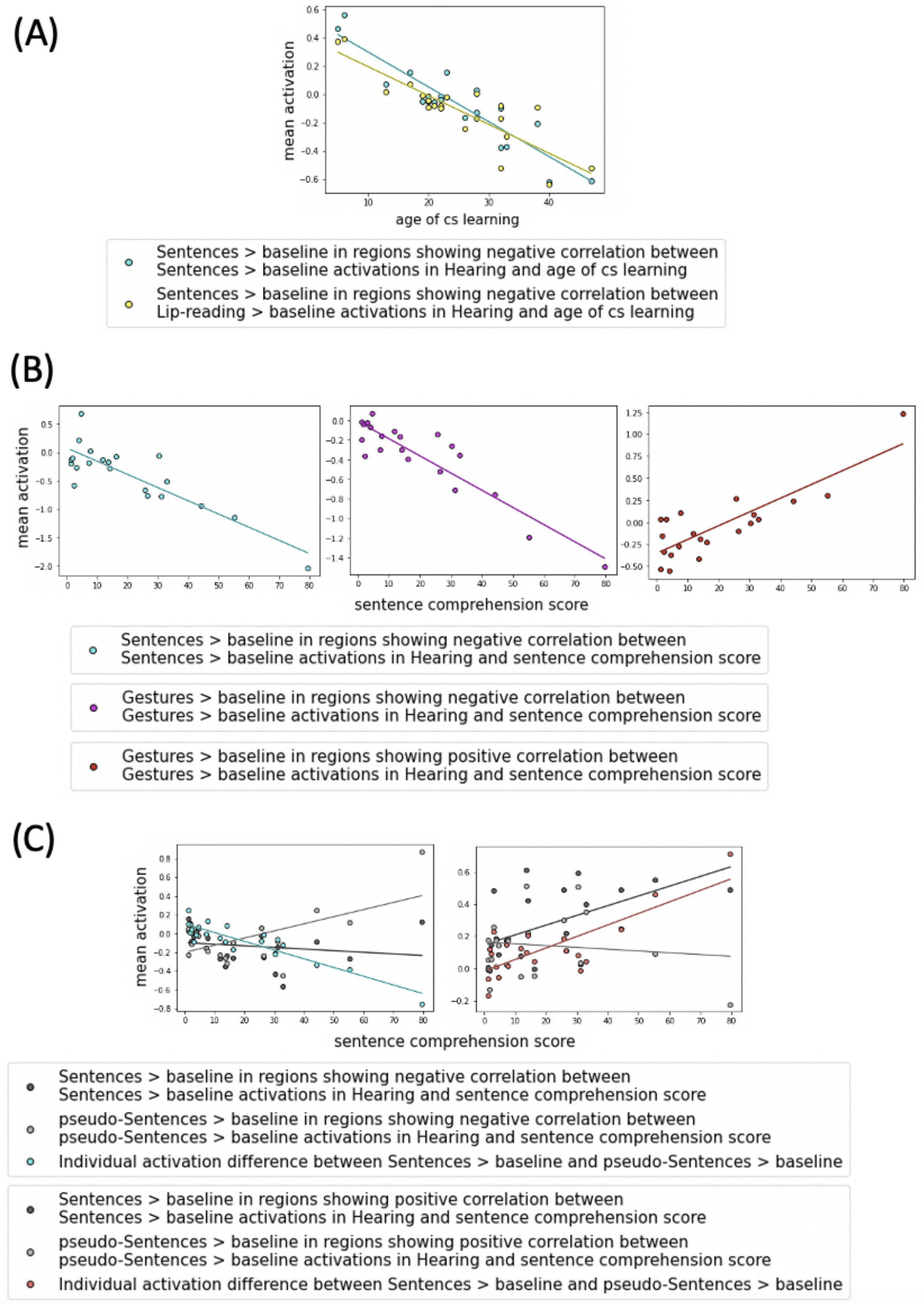

Figure 2 – Plots of individual activation in hearing users, showing the links with the age of CS acquisition and the CS comprehension proficiency. Order of display and color codes are identical to corresponding activations in the above figure 1.

References for the Supplementary materials

Aparicio, M., Peigneux, P., Charlier, B., Balériaux, D., Kavec, M., & Leybaert, J. (2017). The Neural Basis of Speech Perception through Lipreading and Manual Cues: Evidence from Deaf Native Users of Cued Speech. *Frontiers in Psychology*, *8*. https://doi.org/10.3389/fpsyg.2017.00426

Belin, P., Zatorre, R. J., Lafaille, P., Ahad, P., & Pike, B. (2000). Voice-selective areas in human auditory cortex. *Nature*, *403*(6767), 309–312. https://doi.org/10.1038/35002078

Benetti, S., van Ackeren, M. J., Rabini, G., Zonca, J., Foa, V., Baruffaldi, F., Rezk, M., Pavani, F., Rossion, B., & Collignon, O. (2017). Functional selectivity for face processing in the temporal voice area of early deaf individuals. *Proceedings of the National Academy of Sciences*, *114*(31), E6437–E6446. https://doi.org/10.1073/pnas.1618287114

Binder, J. R., Desai, R. H., Graves, W. W., & Conant, L. L. (2009). Where Is the Semantic System? A Critical Review and Meta-Analysis of 120 Functional Neuroimaging Studies. *Cerebral Cortex*, *19*(12), 2767–2796. https://doi.org/10.1093/cercor/bhp055

Brainard, D. H. (1997). The Psychophysics Toolbox. *Spatial Vision*, *10*(4), 433–436. https://doi.org/10.1163/156856897X00357

Chen, X., Affourtit, J., Ryskin, R., Regev, T. I., Norman-Haignere, S., Jouravlev, O., Malik-Moraleda, S., Kean, H., Varley, R., & Fedorenko, E. (2023). The human language system, including its inferior frontal component in "Broca's area," does not support music perception. *Cerebral Cortex*, *33*(12), 7904–7929. https://doi.org/10.1093/cercor/bhad087

Dehaene-Lambertz, G., Monzalvo, K., & Dehaene, S. (2018). The emergence of the visual word form: Longitudinal evolution of category-specific ventral visual areas during reading acquisition. *PLOS Biology*, *16*(3), e2004103. https://doi.org/10.1371/journal.pbio.2004103

Eickhoff, S. B., Stephan, K. E., Mohlberg, H., Grefkes, C., Fink, G. R., Amunts, K., & Zilles, K. (2005). A new SPM toolbox for combining probabilistic cytoarchitectonic maps and functional imaging data. *Neuroimage*, *25*(4), 1325–1335. https://doi.org/10.1016/j.neuroimage.2004.12.034

Emmorey, K., McCullough, S., & Weisberg, J. (2016). The neural underpinnings of reading skill in deaf adults. *Brain and Language*, *160*, 11–20.

Friston, K. J., Penny, W. D., & Glaser, D. E. (2005). Conjunction revisited. *NeuroImage*, *25*(3), 661–667. https://doi.org/10.1016/j.neuroimage.2005.01.013

Glezer, L. S., Weisberg, J., Farnady, C. O., McCullough, S., Midgley, K. J., J Holcomb, P., & Emmorey, K. (2018). Orthographic and phonological selectivity across the reading system in deaf skilled readers. *Neuropsychologia*, *117*, 500–512. https://doi.org/10.1016/j.neuropsychologia.2018.07.010

Graves, W. W., Boukrina, O., Mattheiss, S. R., Alexander, E. J., & Baillet, S. (2017). Reversing the Standard Neural Signature of the Word–Nonword Distinction. *Journal of Cognitive Neuroscience*, *29*(1), 79–94. https://doi.org/10.1162/jocn_a_01022

Hirshorn, E. A., Dye, M. W., Hauser, P. C., Supalla, T., & Bavelier, D. (2022). Reading in Deaf Individuals: Examining the role of visual word form area. *Changing Brains*, 117–137.

Lazard, D. S., & Giraud, A.-L. (2017). Faster phonological processing and right occipito-temporal coupling in deaf adults signal poor cochlear implant outcome. *Nature Communications*, *8*(1), 14872.

https://doi.org/10.1038/ncomms14872

Nichols, T., Brett, M., Andersson, J., Wager, T., & Poline, J.-B. (2005). Valid conjunction inference with the minimum statistic. *NeuroImage*, *25*(3), 653–660. https://doi.org/10.1016/j.neuroimage.2004.12.005

Nichols, T. E., & Holmes, A. P. (2002). Nonparametric permutation tests for functional neuroimaging: A primer with examples. *Human Brain Mapping*, *15*(1), 1–25. https://doi.org/10.1002/hbm.1058

Pasupathy, A., Popovkina, D. V., & Kim, T. (2020). Visual Functions of Primate Area V4. *Annual Review of Vision Science*, *6*, 363–385. https://doi.org/10.1146/annurev-vision-030320-041306

Poldrack, R. A., Desmond, J. E., Glover, G. H., & Gabrieli, J. D. (1998). The neural basis of visual skill learning: An fMRI study of mirror reading. *Cerebral Cortex*, *8*(1), 1–10.

Rottschy, C., Eickhoff, S. B., Schleicher, A., Mohlberg, H., Kujovic, M., Zilles, K., & Amunts, K. (2007). Ventral visual cortex in humans: Cytoarchitectonic mapping of two extrastriate areas. *Human Brain Mapping*, *28*(10), 1045–1059. https://doi.org/10.1002/hbm.20348

Rouger, J., Lagleyre, S., Démonet, J., Fraysse, B., Deguine, O., & Barone, P. (2011). Evolution of crossmodal reorganization of the voice area in cochlear-implanted deaf patients. *Human Brain Mapping*, *33*(8), 1929–1940. https://doi.org/10.1002/hbm.21331

Shamloo, F., & Helie, S. (2016). Changes in default mode network as automaticity develops in a categorization task. *Behavioural Brain Research*, *313*, 324–333. https://doi.org/10.1016/j.bbr.2016.07.029

Smallwood, J., Bernhardt, B. C., Leech, R., Bzdok, D., Jefferies, E., & Margulies, D. S. (2021). The default mode network in cognition: A topographical perspective. *Nature Reviews Neuroscience*, *22*(8), 503–513. https://doi.org/10.1038/s41583-021-00474-4

Thomas Yeo, B. T., Krienen, F. M., Sepulcre, J., Sabuncu, M. R., Lashkari, D., Hollinshead, M., Roffman, J. L., Smoller, J. W., Zöllei, L., Polimeni, J. R., Fischl, B., Liu, H., & Buckner, R. L. (2011). The organization of the human cerebral cortex estimated by intrinsic functional connectivity. *Journal of Neurophysiology*, *106*(3), 1125–1165. https://doi.org/10.1152/jn.00338.2011

Weber, S., Aleman, A., & Hugdahl, K. (2022). Involvement of the default mode network under varying levels of cognitive effort. *Scientific Reports*, *12*(1), 6303. https://doi.org/10.1038/s41598-022-10289-7

Zhan, M., Pallier, C., Agrawal, A., Dehaene, S., & Cohen, L. (2023). Does the visual word form area split in bilingual readers? A millimeter-scale 7-T fMRI study. *Science Advances*, *9*(14), eadf6140.

Supplementary figures

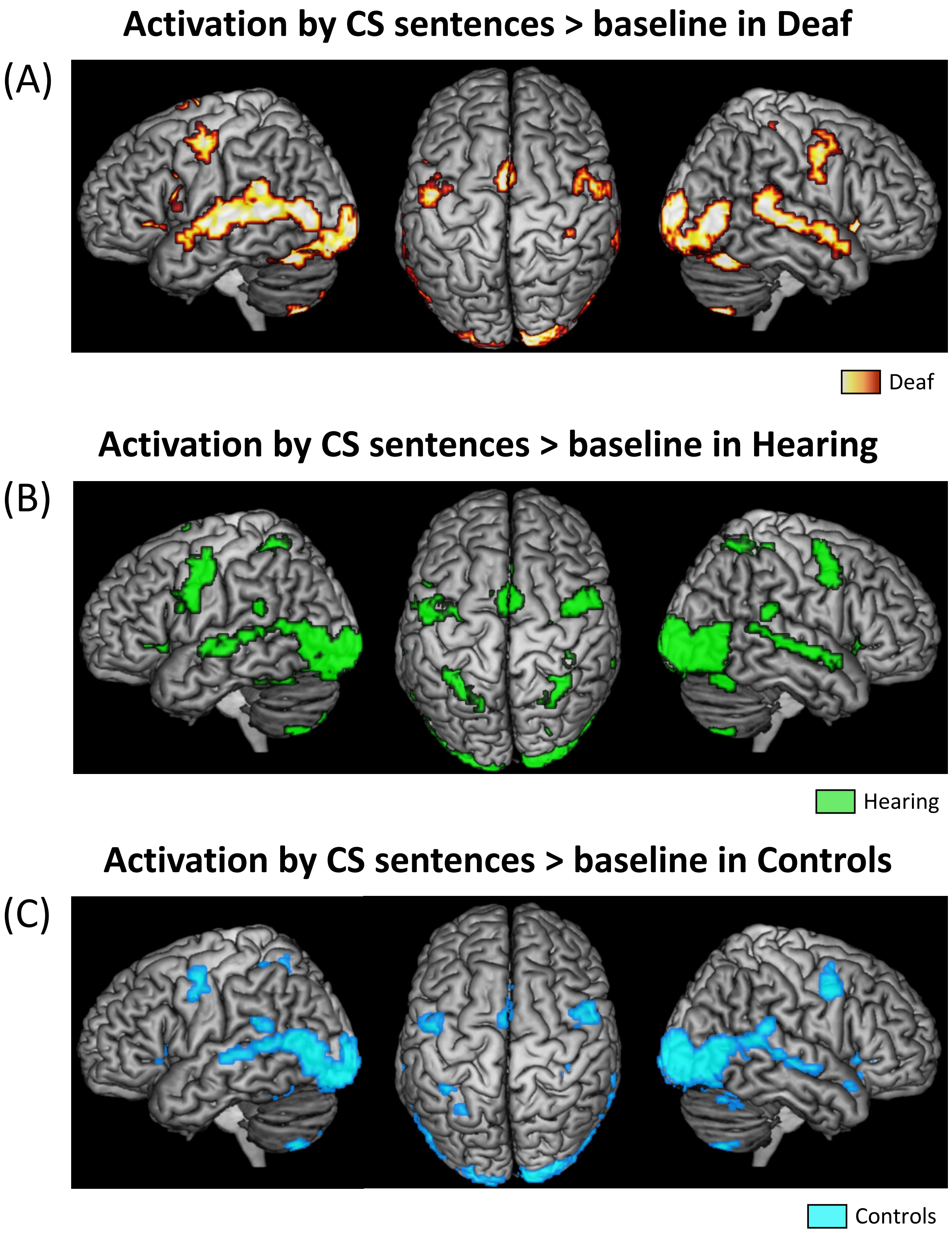

Supplementary figure 1 – Activations by CS sentences > baseline in each group. (A) Activation in deaf users; (B) Activation in hearing users; (C) Activation in controls

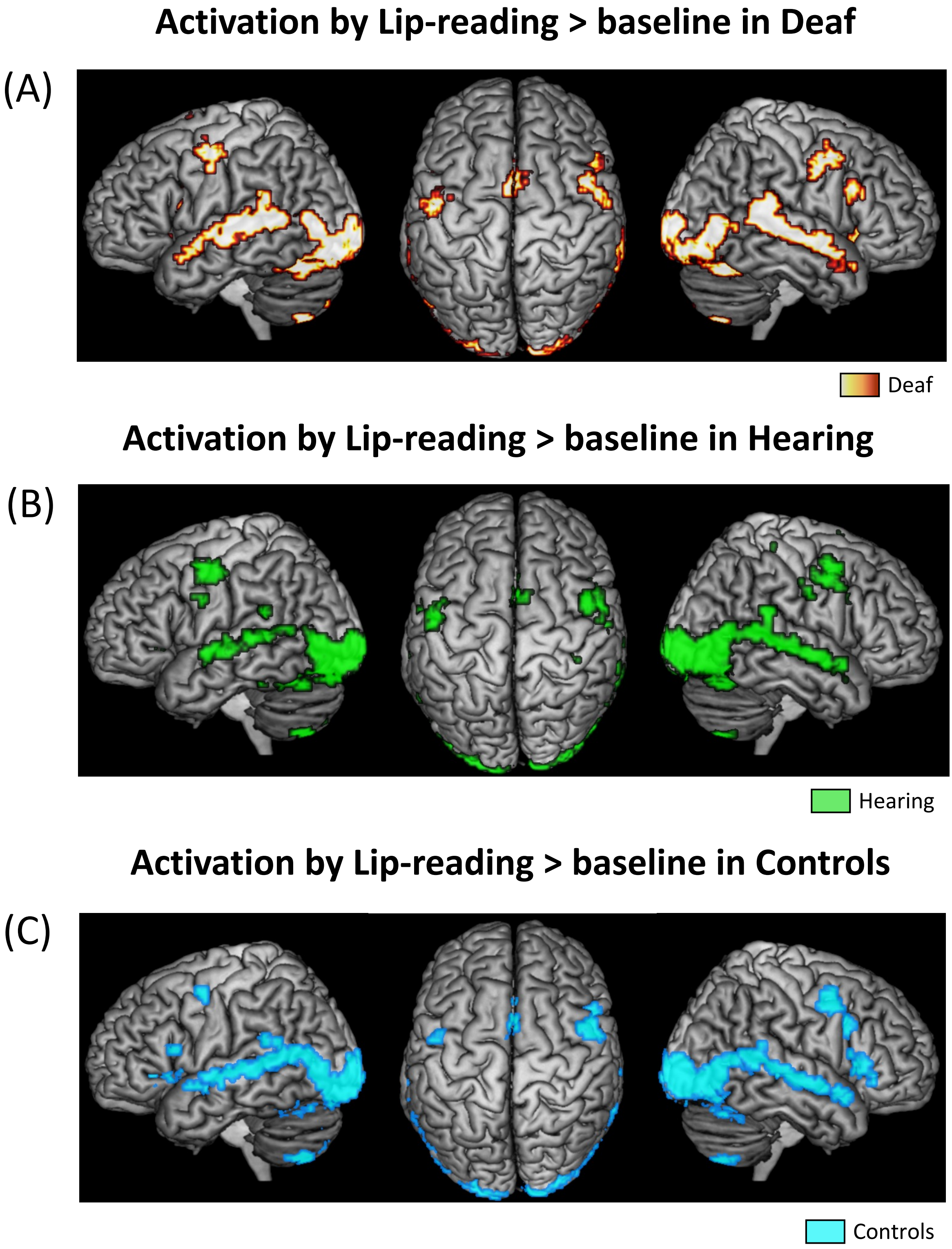

Supplementary figure 2 – Activations by Lip-reading > baseline in each group. (A) Activation in deaf users; (B) Activation in hearing users; (C) Activation in Controls

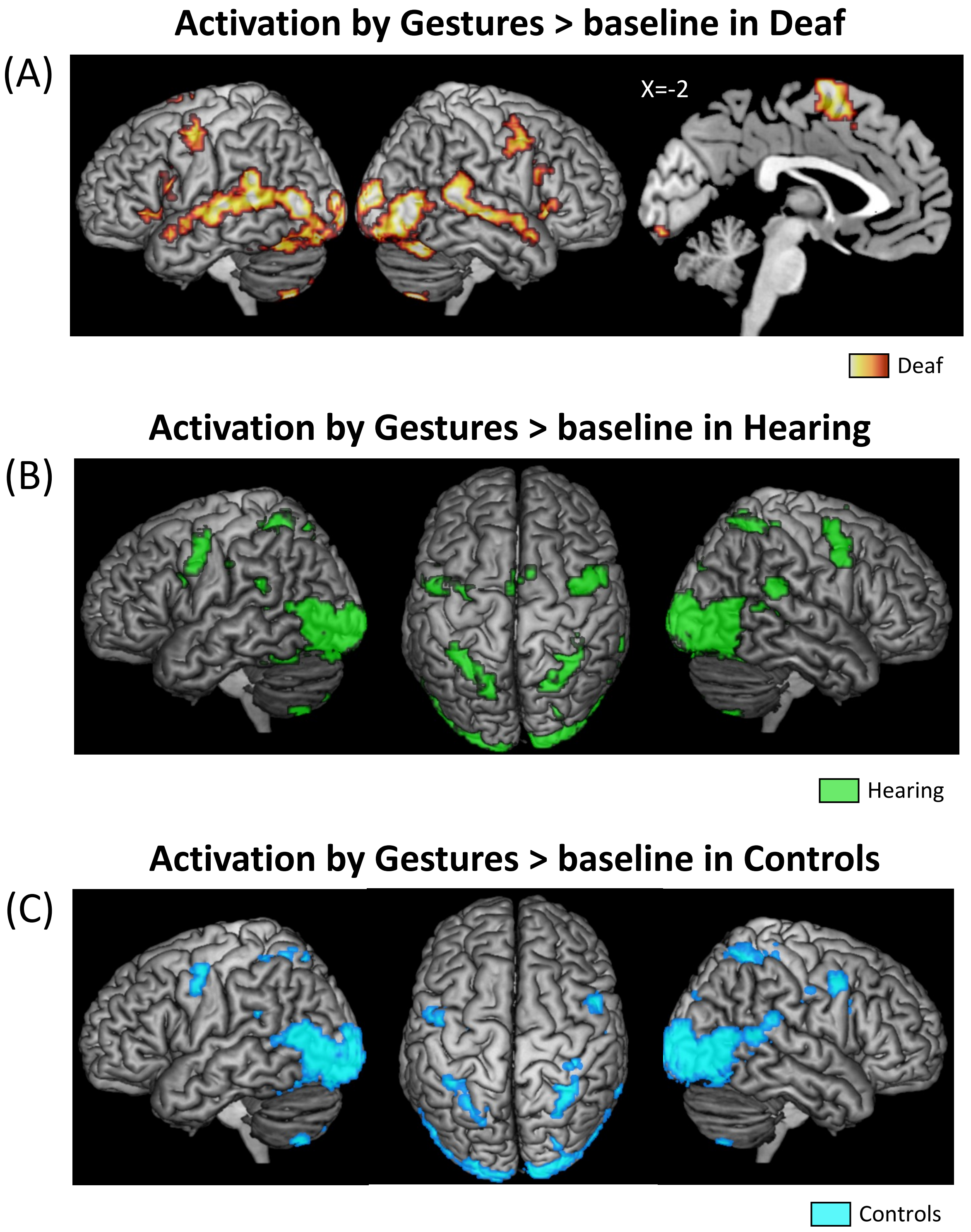

Supplementary figure 3 – Activations by Gestures > baseline in each group. (A) Activation in deaf users; (B) Activation in hearing users; (C) Activation in Controls

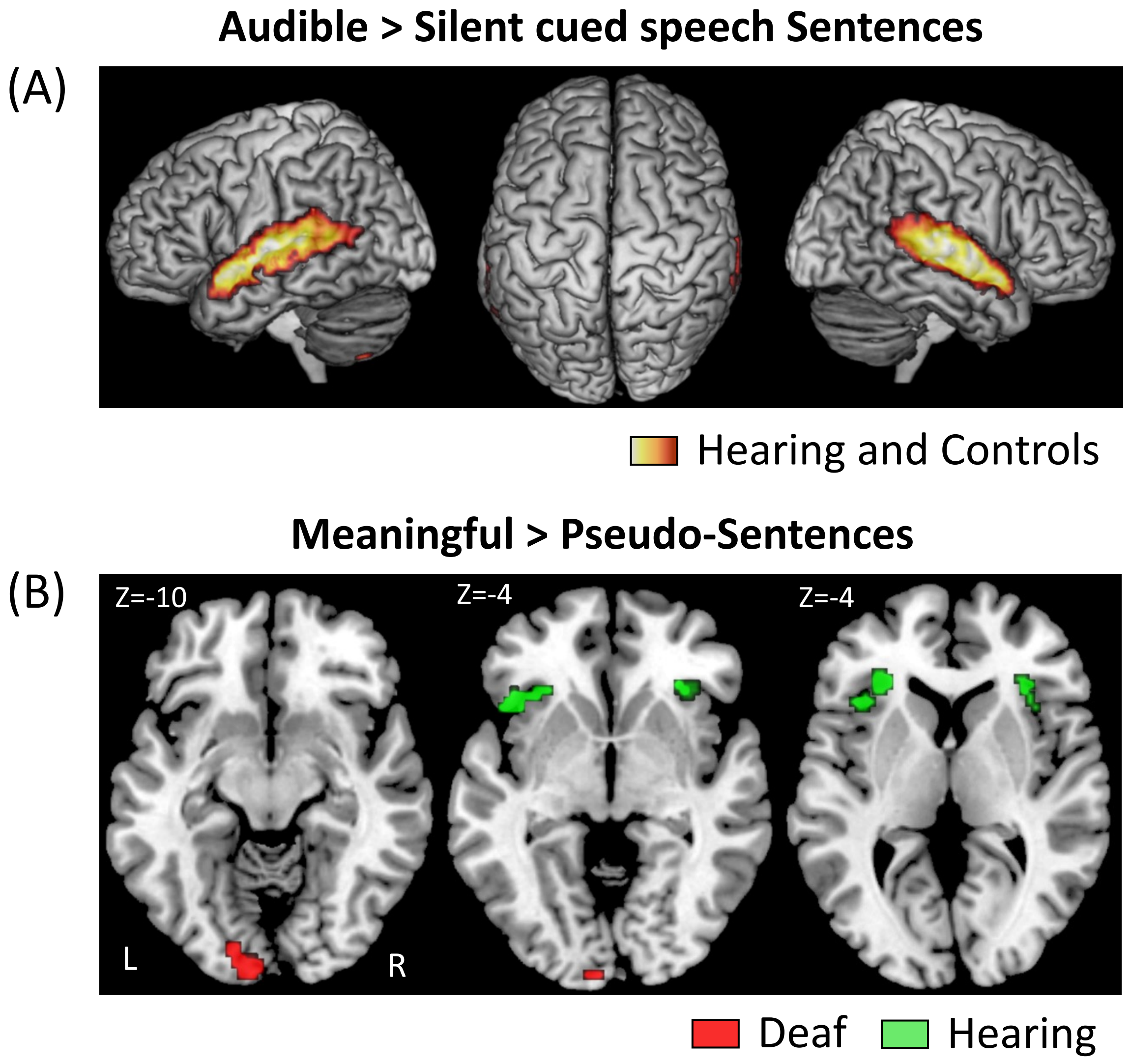

Supplementary figure 4. (A) Activation by Audible > silent Sentences : Activations common to hearing and control participants; (B) Activation by meaningful > Pseudo-sentences in Deaf and Hearing participants.

Supplementary tables

Region	Conjunction 3 groups				Deaf > Controls			
	x	y	z	Z	x	y	z	Z
L Calcarine sulcus	-15	-101	-7	7.4				
R Calcarine sulcus	15	-101	-2	7.18				
L Inferior occipital gyrus	-12	-91	-14	6.33				
R Inferior occipital gyrus	12	-91	-10	6.21				
L Middle occipital gyrus	-20	-96	-2	7.1				
L LOTC	-45	-71	6	5.75				
R LOTC	50	-68	-20	6.73				
L Fusiform gyrus	-42	-51	-22	5.39				
R Fusiform gyrus	42	-48	-20	5.13				
L aSTS/STG	-60	-18	-4	4.04				
R aSTS/STG	50	-38	8	6.51				
L mSTS/STG	-65	-28	3	4.24				
R mSTS/STG	55	-26	0	5.4				
L pSTS/STG	-50	-46	8	5.11	-58	-41	10	4.51
					-45	-38	18	3.65
R pSTS/STG	55	-16	-7	5.37	62	-36	8	4.79
L Precentral gyrus	-42	-1	50	3.56				
R Precentral gyrus	48	2	46	4.39				
L/R SMA	-2	6	58	4.55				
L Cerebellum	-28	-66	-54	5.12				
	-10	-74	-44	4.3				
	-30	-61	-27	4.53				
	-15	-71	-24	4.14				
	-42	-64	-27	3.75				
R Cerebellum	42	-64	-27	4.68				
	32	-61	-24	4.34				
	30	-64	-52	5.29				

Supplementary table 1 - Lip-reading > baseline. Hemisphere and anatomical regions, MNI coordinates and Z score of peak activations. Voxelwise threshold $p<0.001$, clusterwise threshold $p<0.05$ FDR-corrected over the whole brain. Comparisons between groups are masked by the considered activation in the first group. L=left; R=right; a=anterior; m=middle; p=posterior; LOTC=lateral occipitotemporal cortex; STS=superior temporal sulcus; STG=superior temporal gyrus; SMA=supplementary motor area

	Conjunction 3 groups				Deaf				Controls				Deaf > Controls				Deaf > Hearing				Hearing > Deaf			
Region	x	y	z	Z	x	y	z	Z	x	y	z	Z	x	y	z	Z	x	y	z	Z	x	y	z	Z
L Calcarine sulcus	-12	-101	-7	7.37	-15	-98	-2	6.2	-8	-101	-7	6.5												
R Calcarine sulcus	12	-101	3	> 8	10	-101	3	6.78	12	-101	3	6.44												
L Inferior occipital gyrus	-22	-98	3	6.52																	-40	-71	-10	3.63
L Middle occipital gyrus																					-38	-86	0	3.95
R Superior occipital gyrus									28	-71	30	4.19												
L LOTC	-48	-71	3	7.22	-48	-71	3	6.05	-45	-71	3	6.47												
R LOTC	48	-68	-2	> 8	50	-68	-4	6.4	42	-64	3	6.25												
R Fusiform gyrus	42	-56	-17	5.49					42	-48	-22	6.3												
L aSTS/STG					-52	12	-17	4.06					-62	-18	-2	5.62								
R aSTS/STG													60	-16	-4	5.56	60	2	-4	6.04				
L mSTS/STG													-65	-24	6	5.63	-65	-21	3	6.38				
R mSTS/STG					58	-16	-4	5.49					60	-28	0	5.97	60	-16	-4	5.69				
L pSTS/STG					-60	-41	10	6.03					-58	-41	10	5.99	-58	-34	6	5.7				
R pSTS/STG	50	-38	8	4.21	52	-36	3	5.66					62	-36	8	6.11	65	-36	8	5.51				
L MTS/MTG	-48	-44	10	4.03																				
L Supramarginal gyrus	-45	-41	26	4.31					-45	-38	26	4.29												
R Supramarginal gyrus	60	-36	23	4.28																				
L IFG (pars opercularis)					-48	12	16	4.82	48	12	23	3.71	-48	12	13	4.59								
L IFG (pars orbitalis)					-40	24	0	4.41																
R IFG (pars opercularis)					52	16	23	3.97																
R IFG (pars triangularis)					52	19	0	3.81																
L Insula					-30	16	3	4.58					-35	26	-2	3.98	-45	26	-4	3.99				
R Insula					35	22	-2	5.01					35	26	6	4.58								
L Precentral gyrus	-42	-4	50	4.03	-42	-4	53	5.28	-45	-4	50	4.49									-52	4	33	4.18
									-48	-4	23	3.53												
R Precentral gyrus	48	4	43	4.15	45	6	43	5.22	48	6	46	4.36												
R Postcentral gyrus	35	-34	53	4.26	32	-36	50	3.86	35	-34	53	4.93												
									50	-16	36	4.26												
L/R SMA					-2	4	68	6.29					-2	2	68	4.99	-2	4	68	5.09				
L IPS									-18	-56	53	4.73									-38	-36	40	5.35
									-45	-24	38	4.37									-20	-61	60	4.83
R IPS	32	-51	60	4.25					32	-54	63	4.81									15	-64	60	3.66
L FEF									-28	-6	48	4.3									-25	-6	60	4.55
R FEF																								
L Cerebellum	-10	-74	-44	5	-25	-61	-44	5.26	-10	-76	-44	5.32												
					-10	-81	-44	4.24	-28	-64	-50	4.36												
					-15	-74	-22	4.85																
R Cerebellum	12	-76	-44	4.57	28	-66	-57	5.98	12	-76	-47	4.58	28	-66	-54	5.33	25	-66	-50	3.98				
					15	-74	-40	4.24	28	-66	-54	4.32												
					18	-74	-22	4.81	15	-66	-27	3.76	32	-61	-24	5.49	32	-61	-24	5.1				

Supplementary table 2 - Gestures > baseline. Hemisphere and anatomical regions, MNI coordinates and Z score of peak activations. Voxelwise threshold $p<0.001$, clusterwise threshold $p<0.05$ FDR-corrected over the whole brain. Comparisons between groups are masked by the considered activation in the first group. L=left; R=right; a=anterior; p=posterior; LOTC=lateral occipitotemporal cortex; STS=superior temporal sulcus; STG=superior temporal gyrus; MTS=middle temporal sulcus; MTG=middle temporal gyrus; IFG=inferior frontal gyrus; SMA=supplementary motor area; IPS=intraparietal sulcus; FEF=frontal eye field

Region	Deaf				Hearing				Controls				Deaf > Controls				Hearing > Deaf				Controls > Deaf			
	x	y	z	Z	x	y	z	Z	x	y	z	Z	x	y	z	Z	x	y	z	Z	x	y	z	Z
L LOTC	-32	-94	-7	4.64	-30	-91	-10	4.38	-32	-94	-12	4.3	-45	-71	3	4.24								
R LOTC					32	-94	-7	5.3																
L aSTS/STG					-55	-4	-10	5.69	-60	-8	-4	5.78					-60	-6	-4	6.01	-58	-11	-4	5.73
R aSTS/STG					55	-8	-10	5.87	58	-4	-10	5.17					48	-31	6	4.98	60	-14	-7	5.8
L mSTS/STG					-65	-21	-2	5.56	-60	-28	0	5.31					-60	-34	3	5.66	-62	-34	3	5.72
R mSTS/STG					65	-26	0	5.67	60	-11	-4	5.3					62	-21	-2	5.4	45	-24	-2	4.72
L pSTS/STG									-62	-41	3	5.34					-48	-46	8	5.73				
L pSTG/SMG																					-62	-44	18	4.07
R pSTS/STG									60	-31	0	5.77					55	-8	-10	5.69	55	-34	3	5.09
L IFG (pars triangularis)									-48	12	0	4.51									-35	29	-2	6.07
L IFG (pars orbitalis)									-38	32	0	4.71												
R IFG (pars triangularis)									45	24	0	4.46									50	19	-2	4.17
L Precentral gyrus					-55	-11	43	4.09									-52	-8	43	4.33				
L/R SMA									2	4	60	4.81					0	9	58	4.78	0	4	63	5.21
L Cerebellum																	-45	-66	-27	4.47				
									-30	-64	-52	3.91									-25	-76	-50	3.99
R Cerebellum																	45	-64	-27	4.85	32	-61	-27	4.5
					30	-66	-52	4.14	30	-64	-50	4.26					28	-66	-50	5.97	28	-66	-50	6.07

Supplementary table 3 - Lip-reading > Gestures. Hemisphere and anatomical regions, MNI coordinates and Z score of peak activations. Voxelwise threshold $p<0.001$, clusterwise threshold $p<0.05$ FDR-corrected over the whole brain. Comparisons between groups are masked by the considered activation in the first group. L=left; R=right; a=anterior; m=middle; p=posterior; LOTC=lateral occipitotemporal cortex; STS=superior temporal sulcus; STG=superior temporal gyrus; SMG=supramarginal gyrus; IFG=inferior frontal gyrus; SMA=supplementary motor area

	Deaf				Hearing				Controls				Deaf > Controls				Deaf > Hearing				Controls > Deaf			
**Region**	**x**	**y**	**z**	**Z**	**x**	**y**	**z**	**Z**	**x**	**y**	**z**	**Z**	**x**	**y**	**z**	**Z**	**x**	**y**	**z**	**Z**	**x**	**y**	**z**	**Z**
L Calcarine sulcus	-10	-101	0	4.3					-5	-101	3	3.77												
R Calcarine sulcus	10	-101	3	6.26	12	-98	8	5.89																
R Middle occipital gyrus	20	-96	13	6.04																				
L Superior occipital gyrus					-22	-74	33	5.52																
R Superior occipital gyrus					28	-74	33	4.88	25	-78	33	4.55												
L LOTC	-48	-71	3	5.73	-48	-71	0	6.07	-45	-71	3	6.38									-38	-84	-2	4.85
																					-45	-71	3	4.24
R LOTC	48	-66	3	6.27	50	-68	-2	5.79	52	-66	-2	6.01												
L LOT sulcus	-45	-44	-22	4.44	-40	-64	-2	5.31																
R LOT sulcus					42	-54	-12	3.75																
L Fusiform gyrus									-40	-51	-14	3.94												
R Fusiform gyrus									-15	-88	-12	4.96												
L aSTS/STG													-58	-11	-4	5.73	-60	-6	-4	6.01				
R aSTS/STG													60	-14	-7	5.8	60	-11	-10	5.47				
L mSTS/STG													-62	-34	3	5.72	-60	-34	3	5.66				
R mSTS/STG													45	-24	-2	4.72	62	-21	-2	5.4				
L pSTS/STG	-50	-46	8	4.68													-48	-46	8	5.73				
																	-58	-38	18	4.03				
R pSTS/STG	60	-34	18	4.37	65	-41	18	3.91					55	-34	3	5.09	48	-31	6	4.98				
L Supramarginal gyrus	-55	-41	23	5.23																				
R Supramarginal gyrus					55	-34	26	4.72																
L IFG (pars opercularis)	-48	6	13	4.13									-48	12	10	4.48	-52	12	18	3.99				
	-38	6	26	3.98																				
L IFG (pars triangularis)	-35	29	-2	3.95									-35	29	-2	6.07								
R IFG (pars orbitalis)																								
L Insula													-30	22	8	4.85	-38	29	-2	4.07				
R Insula													38	26	8	4.92	38	26	8	4.14				
L Precentral gyrus	-42	-1	50	4.43	-52	2	33	5									-52	-8	43	4.33				
R Postcentral gyrus	32	-36	48	3.82					32	-36	53	4.73												
L SMA	-5	2	68	4.52																				
L/R SMA													0	4	63	5.21	0	9	58	4.78				
R Middle frontal gyrus	40	-4	56	4.25																				
L IPS					-35	-41	46	6.32	-18	-58	58	5.7												
					-22	-64	60	7.06	-25	-58	63	4.46												
R IPS					30	-48	56	5.32																
L FEF					-25	-11	53	5.67																
R FEF					40	-6	50	4.71																
L Cerebellum													-28	-66	-57	3.74								
																	-45	-66	-27	4.47				
R Cerebellum	28	-66	-50	4.72									28	-66	-50	6.07	28	-66	-50	5.97				
	45	-58	-30	4.91									45	-64	-27	4.77	48	-61	-30	5.16				
	32	-61	-27	3.96									32	-61	-27	4.5								

Supplementary table 4 - Gestures > Lip-reading. Hemisphere and anatomical regions, MNI coordinates and Z score of peak activations. Voxelwise threshold p<0.001, clusterwise threshold p<0.05 FDR-corrected over the whole brain. Comparisons between groups are masked by the considered activation in the first group. L=left; R=right; a=anterior; m=middle; p=posterior; LOTC=lateral occipitotemporal cortex; STS=superior temporal sulcus; STG=superior temporal gyrus; IFG=inferior frontal gyrus; SMA=supplementary motor area; IPS=intraparietal sulcus; FEF=frontal eye field

Contrast	Deaf				Hearing				Control				Deaf > Controls				Deaf > Hearing				Hearing > Controls			
	Left		Right		Left		Right		Left		Right		Left		Right		Left		Right		Left		Right	
	B10	d	B10	d	B10	d	B10	d	B10	d	B10	d	B10	d	B10	d	B10	d	B10	d	B10	d	B10	d
**CS Sentences > baseline**	2.14	0.73	0.31	0.01	1.33	0.6	0.47	0.32	1.30	0.61	0.34	0.15	7.27	0.94	0.32	0.09	0.32	0.08	0.35	0.17	3.55	0.78	0.32	0.10
**Gestures > baselline**	28.45	1..15	0.87	0.52	1.14	0.57	1.03	0.54	0.48	0.33	1.87	0.68	2.03	0.71	0.31	0.03	0.32	0.11	0.31	0.03	0.55	0.37	0.31	0.06
**Lip-reading > baseline**	0.52	0.37	0.36	0.18	0.51	0.35	0.46	0.32	0.33	0.13	0.36	0.19	0.53	0.37	0.41	0.26	0.32	0.07	0.53	0.36	0.54	0.37	0.32	0.10

Supplementary table 5 – BF_{10} values and Cohen's d in the Visual Word Form Area (VWFA) and in its right homologue. BF_{10} were computed on individual mean activations in spheres centered at the peak value of the word-specific contrast of the functional localizer, voxelwise threshold p<0.001, clusterwise threshold p<0.05 FDR-corrected over the whole brain.

Region	Conjunction Controls & Hearing			
	x	y	z	Z
L STS/STG and MTG	-55	-18	6	> 8
R STS/STG and MTG	52	-14	6	7.74
L Cerebellum	-18	-68	-54	5.11
R Cerebellum	15	-71	-54	4.69

Supplementary table 6 - Audible > Silent Sentences. Hemisphere and anatomical regions, MNI coordinates and Z score of peak activations. Voxelwise threshold p<0.001, clusterwise threshold p<0.05 FDR-corrected over the whole brain. L=left; R=right; STS=superior temporal sulcus; STG=superior temporal gyrus; MTG=middle temporal gyrus

Region	Deaf				Hearing			
	x	y	z	Z	x	y	z	Z
L Lingual gyrus	-8	-96	-10	4.26				
L IFG					-40	22	3	4.75
L Insula					-30	32	3	4.27
R Insula					32	29	6	4.01

Supplementary table 7 - Silent meaningful > Pseudo-Sentences. Hemisphere and anatomical regions, MNI coordinates and Z score of peak activations. Voxelwise threshold p<0.001, clusterwise threshold p<0.05 FDR-corrected over the whole brain. Other groups and comparisons were non-significant. L=left; R=right; IFG=inferior frontal gyrus

(A)

Region	x	y	z	Z
**Words > other categories**				
L Calcarine sulcus	-10	-91	-2	4.72
R Calcarine sulcus	15	-86	-2	4.44
L lateral occipitotemporal sulcus	-50	-58	-17	3.88
L pSTS/STG	-60	-31	-2	3.94
L IFG (pars opercularis)	-40	6	26	3.96
**Faces > other categories**				
L/R Calcarine sulcus	2	-84	-10	4.80
R Cuneus	10	-96	8	4.59
L Fusiform gyrus	-42	-56	-22	4.97
R Fusiform gyrus	42	-51	-20	4.97
R pSTS/STG	52	-44	13	4.08
**Bodies > other categories**				
L LOTC	-48	-78	6	5.45
R LOTC	50	-71	6	6.93

(B)

Region	x	y	z	Z
**Tools > other categories**				
R Middle occipital gyrus	35	-81	6	7.01
R Superior occipital gyrus	28	-76	33	4.63
L LOTC	-30	-88	13	6.8
R LOTC	48	-64	-4	5.7
L Collateral sulcus	-22	-68	-12	6.49
R Collateral sulcus	25	-64	-10	5.51
	30	-68	-14	5.74
L IPS	-25	-56	58	4.07
R IPS	30	-56	63	4.71
**Houses > other categories**				
L Occipital pole	-25	-86	20	6.72
R Occipital pole	15	-96	3	7.12
L Collateral sulcus	-22	-76	-12	7.16
R Collateral sulcus	25	-78	-7	6.17
L Lingual gyrus	-22	-81	-17	7.01
R Lingual gyrus	18	-84	-12	7.34
R Precuneus	20	-56	10	4.95

Supplementary table 8 - Category-specific activations for the conjunction of all three groups. Hemisphere and anatomical regions, MNI coordinates and Z score of peak activations for (A) Words, Faces and Bodies and (B) Tools and Houses. Voxelwise threshold p<0.001, clusterwise threshold p<0.05 FDR-corrected over the whole brain. No comparison showed significant activation. L=left; R=right; p=posterior; STS=superior temporal sulcus; STG=superior temporal gyrus; IFG=inferior frontal gyrus; LOTC=lateral occipitotemporal cortex; IPS=intraparietal sulcus

	Region															
	**L Collateral sulcus**				**L pSTS/STG**				**R pSTS/STG**				**R mSTS/STG**			
	**x**	**y**	**z**	**X**	**x**	**y**	**z**	**X**	**x**	**y**	**z**	**X**	**x**	**y**	**z**	**X**
Words > baseline									65	-36	8	6.12	65	-16	-2	6.11
Faces > baseline	-22	-68	-2	4.43	-58	-44	10	4.48	62	-36	8	6.53	65	-16	-2	5.44
Bodies > baseline	-22	-68	-2	4.37					62	-34	8	6.08				
Tools > baseline									68	-36	8	6.07				

Supplementary table 9 – Comparison Deaf > Hearing + Controls (masked by Deaf) for each category > baseline. Hemisphere and anatomical regions, MNI coordinates and Z score of peak activations. Voxelwise threshold p<0.001, clusterwise threshold p<0.05 FDR-corrected over the whole brain. No Houses > baseline comparison showed significant activation. L=left; R=right; p=posterior; m=middle; STS=superior temporal sulcus; STG=superior temporal gyrus
